# Immune-inflammatory prediction of PICC-related bloodstream infection in oldest-old adults with exploratory berberine biofilm–inflammation experiments

**DOI:** 10.3389/fphar.2026.1914929

**Published:** 2026-08-18

**Authors:** Xia Li, Fulan Chen, Cuixia Zheng, Suli Yin

**Affiliations:** Outpatient Department, General Hospital of Eastern Theater Command, Nanjing, Jiangsu, China

**Keywords:** berberine, biofilm, catheter-related bloodstream infection, competing-risk model, C-reactive protein-to-albumin ratio, immune-inflammatory biomarkers, oldest-old adults, peripherally inserted central catheter

## Abstract

**Background:**

Adults aged 80 years or older frequently require peripherally inserted central catheter (PICC) placement for prolonged intravenous therapy, nutritional support, and difficult vascular access. In this oldest-old population, PICC-related bloodstream infection (PICC-CRBSI) risk may be shaped by catheter factors, frailty, nutritional depletion, immune-inflammatory imbalance, and competing events such as death or non-infectious catheter removal. This study developed and internally validated a competing-risk model for 60-day definite PICC-CRBSI in patients aged ≥80 years and separately explored the antibiofilm and anti-inflammatory activity of berberine *in vitro*.

**Methods:**

Consecutive patients aged ≥80 years who underwent PICC placement at a single tertiary teaching hospital between January 2019 and December 2025 were included in a retrospective cohort. The prediction landmark was 24 h after PICC insertion. Definite PICC-CRBSI within 60 days was analyzed with death and non-infectious PICC removal treated as competing events. Clinical, biomarker, and combined models were developed using L2-penalized Fine–Gray regression and internally validated with 1,000 patient-level bootstrap resamples. After model locking, a separate exploratory substudy evaluated berberine effects on *Staphylococcus* epidermidis biofilms formed on PICC material and on macrophage inflammatory responses induced by risk-stratified donor plasma and sterile biofilm-conditioned medium.

**Results:**

A total of 386 patients were analyzed, and 46 developed definite PICC-CRBSI over 19,528 catheter-days, corresponding to an incidence density of 2.36 per 1,000 catheter-days. Patients with definite PICC-CRBSI had greater frailty, longer catheter dwell time, lower lymphocyte and albumin levels, and higher systemic immune-inflammation index (SII) and C-reactive protein-to-albumin ratio (CAR) than those without definite infection. The combined model showed an apparent 60-day time-dependent area under the curve (AUC) of 0.758 and a bootstrap-corrected AUC of 0.731, indicating moderate internally validated discrimination. The Aalen–Johansen 60-day cumulative incidence of PICC-CRBSI was 3.2%, 8.5%, and 28.1% in the low-, intermediate-, and high-risk groups, respectively. *In vitro*, berberine reduced viable S. epidermidis biofilm burden from 7.656 log_10_ CFU/coupon under vehicle exposure to 6.919 log_10_ CFU/coupon at one-half of the strain-specific minimum inhibitory concentration. In macrophage experiments, high-risk donor plasma induced higher interleukin-6 release than low-risk donor plasma under vehicle conditions, while 10 μM berberine reduced interleukin-6 by approximately 23% without substantial cytotoxicity.

**Conclusion:**

A single-center competing-risk model combining catheter-related variables with routine immune-inflammatory biomarkers provided preliminary internal risk stratification for 60-day PICC-CRBSI in patients aged ≥80 years. The berberine substudy suggested antibiofilm and anti-inflammatory activity *in vitro*, but it did not directly validate the clinical prediction model or establish clinical efficacy. Before the locked prediction equation can be used clinically, independent multicenter validation is required across settings with different PICC products, patient case-mix, catheter-maintenance protocols, blood-culture practices, and local microbiological epidemiology. Further mechanistic studies are also required before berberine-based catheter-infection prevention can be considered.

## 1 Introduction

Peripherally inserted central catheters (PICCs) provide reliable long-term central venous access for patients requiring prolonged antimicrobial therapy, chemotherapy, parenteral nutrition, and other continuous intravenous interventions. Compared with repeated peripheral venipuncture, PICC placement reduces procedural trauma and enables treatment continuity across inpatient and outpatient settings. Nevertheless, PICCs carry risks of bloodstream infection, venous thrombosis, catheter occlusion, and mechanical damage (Safdar and Maki, 2005). Prospective multicenter studies have shown that PICC-related complications vary with treatment setting, catheter dwell time, lumen number, and patient characteristics (Grau et al., 2017; Lee et al., 2019). PICCs are commonly used in older adults with frailty, cognitive impairment, inadequate oral intake, or difficult vascular access (Lafuente et al., 2013). However, patients aged ≥80 years should not be treated simply as an older subset of general adult or younger geriatric cohorts. This oldest-old group has a higher burden of frailty, malnutrition, multimorbidity, immune senescence, care dependency, and competing mortality, all of which may change both infection risk and the interpretation of catheter-related outcomes. In this study, the ≥80-year threshold was selected *a priori* to define a very advanced-age PICC population in whom prediction must account for clinical vulnerability and competing events. The transportability of the model to younger elderly populations remains uncertain and requires separate validation.

PICC-related bloodstream infection (PICC-CRBSI) arises from the interplay of host immune susceptibility, catheter characteristics, infusion regimens, insertion conditions, post-insertion catheter care, microbial contamination, and diagnostic ascertainment. Parenteral nutrition, immunosuppression, chemotherapy, multi-lumen catheters, and prolonged dwell time are established risk factors (Cao et al., 2025). Recent catheter-infection prediction studies and reviews have improved risk estimation by incorporating demographic, catheter-related, treatment-related, and laboratory variables, but most existing models have focused on broader adult, oncology, intensive-care, neonatal, hemodialysis, or mixed central-line populations rather than oldest-old PICC recipients specifically (Asokan et al., 2026a; Fulop et al., 2018). Many models also use binary infection outcomes or conventional survival approaches, whereas death and non-infectious catheter removal may preclude subsequent PICC-CRBSI in very advanced-age cohorts. A landmark-based competing-risk framework is therefore more appropriate for estimating absolute 60-day infection risk in this population.

Advanced aging is accompanied by immunosenescence and chronic inflammatory remodeling, characterized by impaired innate and adaptive immune function, reduced lymphocyte diversity, and persistent activation of systemic inflammatory pathways (Sadighi Akha, 2018; Zhao et al., 2025). These changes weaken host resistance to microbial invasion and sustain a chronic inflammatory baseline (Asokan et al., 2026b). Healthcare-associated infection risk is further shaped by invasive devices, antimicrobial exposure, patient susceptibility, diagnostic timing, and local microbiological ecology. Routine hematological and biochemical indices can capture several clinically relevant aspects of this phenotype. The systemic immune-inflammation index (SII) integrates peripheral neutrophil, lymphocyte, and platelet counts, whereas the C-reactive protein-to-albumin ratio (CAR) reflects the balance between inflammatory activity and nutritional reserve. Procalcitonin may add information about bacterial inflammatory activity, but its use in retrospective data is vulnerable to selective testing and missingness. For that reason, this study evaluated SII, CAR, and procalcitonin as routine immune-inflammatory predictors while separately assessing the influence of missing procalcitonin data. NLR, PLR, frailty, ICU/HDU care, baseline infection, recent antimicrobial exposure, and malignancy were retained for descriptive, comparative, or sensitivity analyses rather than being forced into the primary combined model, because the number of definite PICC-CRBSI events limited allowable model complexity.

Biofilm formation is a central mechanism of persistent catheter-related infection. After adhering to catheter surfaces, microorganisms proliferate within an extracellular matrix, reduce antimicrobial penetration, evade host immune clearance, and sustain local inflammatory stimulation. Contemporary reviews of medical-device biofilm infections emphasize that biofilm pathogenesis involves microbial adhesion, matrix maturation, antimicrobial tolerance, diagnostic difficulty, and incomplete eradication by standard antimicrobial therapy (Asokan et al., 2026c). Coagulase-negative staphylococci, especially *Staphylococcus* epidermidis, are frequent catheter-associated organisms and are well suited for exploratory biofilm experiments because of their strong surface-adherence and biofilm-forming capacity. However, PICC-CRBSI is clinically heterogeneous and may involve Gram-positive bacteria, Gram-negative bacteria, and fungi; therefore, experiments using S. epidermidis alone cannot represent the full microbial spectrum of PICC-CRBSI. Berberine, a plant-derived isoquinoline alkaloid, has been reported to modulate macrophage inflammatory signaling through AMP-activated protein kinase and nuclear factor kappa B (NF-κB)-related pathways (Mo et al., 2014). Subinhibitory berberine exposure has also been associated with altered staphylococcal biofilm formation and maturation (Wang et al., 2009). These findings provide a biological rationale for an exploratory *in vitro* substudy, but they do not establish that berberine is clinically achievable, clinically effective, or able to validate a clinical prediction model.

This study had two linked but analytically distinct components. The first component developed and internally validated a 60-day competing-risk prediction model for definite PICC-CRBSI in patients aged ≥80 years, using clinical and biomarker data available at a 24-h post-insertion landmark. The second component was a separate exploratory translational substudy that evaluated whether strain-specific subinhibitory berberine exposure reduced S. epidermidis biofilm formation on polyurethane PICC material and attenuated macrophage inflammatory responses induced by risk-stratified donor plasma and sterile biofilm-conditioned medium. The primary clinical hypothesis was that a combined catheter-related and immune-inflammatory model would provide better internally validated 60-day risk stratification than clinical or biomarker information alone under competing-risk conditions. The exploratory experimental hypothesis was that berberine would reduce S. epidermidis biofilm burden and macrophage inflammatory activation *in vitro* without substantial cytotoxicity.

## 2 Materials and methods

### 2.1 Study design, setting, and ethics

This single-center study comprised two sequential components: a retrospective cohort study for the development and internal validation of a competing-risk prediction model for definite PICC-related bloodstream infection (PICC-CRBSI), and a separate exploratory translational substudy evaluating berberine-related biofilm and inflammatory responses *in vitro*. The two components were linked by the immune-inflammatory risk-stratification framework but were analyzed as distinct components; the translational substudy was not designed to validate the clinical prediction model or establish clinical efficacy of berberine.

The retrospective cohort enrolled consecutive patients aged ≥80 years who underwent PICC implantation at a tertiary teaching hospital in China from January 1, 2019 to December 31, 2025. Follow-up was extended to March 2, 2026 to ensure that all eligible patients completed the predefined 60-day observation window. The ≥80-year threshold was selected *a priori* to define an oldest-old PICC population with high frailty burden, frequent nutritional-support needs, multimorbidity, immune-inflammatory vulnerability, and a clinically relevant probability of death or non-infectious catheter removal before infection occurrence. Clinical data cleaning, outcome adjudication, multiple imputation, model construction, and internal validation were completed before model locking. Prospective plasma collection and translational experiments were conducted at the same institution from March 15 to May 31, 2026. No prospective donor was stratified using interim or revised model versions.

**FIGURE 1:**
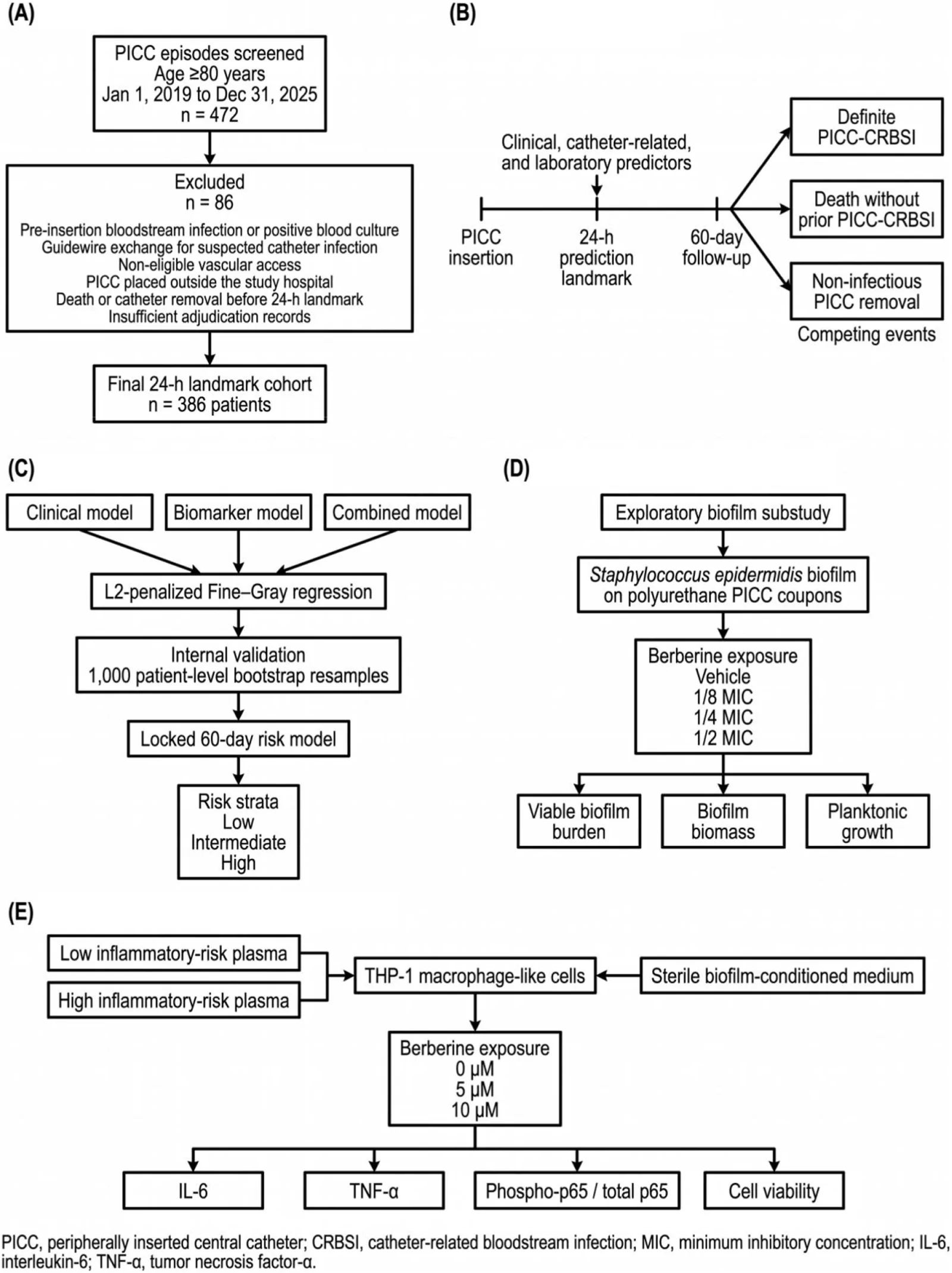
Study design, patient selection, and analytical workflow. **(A)** Consecutive patients aged ≥80 years undergoing PICC placement were screened. Of 472 PICC episodes, 86 were excluded for pre-insertion bloodstream infection or positive blood culture, guidewire exchange for suspected catheter infection, non-eligible vascular access, outside-hospital PICC placement, death or catheter removal before the 24-h landmark, or insufficient adjudication records, leaving 386 patients in the landmark cohort. **(B)** Follow-up started at the 24-h landmark and continued for 60 days, with death and non-infectious PICC removal treated as competing events. **(C)** Clinical, biomarker, and combined Fine–Gray models were developed, internally validated, and locked before donor screening. **(D)** The exploratory substudy tested berberine effects on *Staphylococcus* epidermidis biofilms on polyurethane PICC coupons. **(E)** THP-1 macrophage-like cells were exposed to risk-stratified donor plasma, sterile biofilm-conditioned medium, and berberine. PICC, peripherally inserted central catheter; CRBSI, catheter-related bloodstream infection.

The 24-h post-insertion time point served as the unified prediction landmark for all predictive analyses. Only clinical, catheter-related, and laboratory data acquired within 24 h of PICC insertion were considered as candidate predictors. The primary endpoint was the cumulative incidence of definite PICC-CRBSI within 60 days after the landmark, with all-cause death without prior PICC-CRBSI and non-infectious PICC removal treated as competing events. The 24-h landmark was used to avoid including patients who died or had catheter removal immediately after insertion and to ensure that all predictors reflected baseline or early post-insertion status rather than evolving infection. The study workflow and patient-selection process are illustrated in Figure 1. Reporting followed the STROBE statement and TRIPOD guidance for clinical prediction models (von Elm et al., 2007; Collins et al., 2015).

The study protocol was approved by the institutional ethics committee. The hospital name and ethics approval number were masked for blinded peer review. For the retrospective component, individual informed consent was waived owing to the use of de-identified routine clinical data. For the prospective translational component, written informed consent for blood collection, storage, and *in vitro* experimental use was obtained from all participants or their legally authorized representatives. All data used for modeling were de-identified before analysis. The translational substudy used prospectively collected plasma and unused sterile PICC materials; no experimental intervention was administered to patients.

### 2.2 Clinical cohort, PICC management, and follow-up

All potential subjects were screened from the institutional vascular access registry, with data integrated from electronic medical records, clinical laboratory databases, microbiology systems, nursing documentation, catheter maintenance records, emergency department records, and readmission records. Inclusion criteria were: age ≥80 years at PICC insertion, newly placed upper-extremity PICC at the study hospital, survival with an intact catheter at the 24-h landmark, and sufficient data for eligibility determination, catheter course tracking, follow-up, and outcome evaluation. Missing predictor data did not cause exclusion and were handled by prespecified imputation procedures.

To preserve observational independence, only the first eligible PICC episode per patient was included. Exclusion criteria were: confirmed bloodstream infection or positive blood culture within 48 h before PICC insertion; guidewire exchange for suspected catheter infection; prior tunneled central venous catheter, implanted port, midline catheter, or lower-extremity PICC; PICC insertion outside the study hospital; death or catheter removal before the 24-h landmark; and insufficient clinical or microbiological data for outcome adjudication. All consecutive eligible patients were included without case sampling, outcome-based enrichment, or matching. The number of screened episodes and the reasons for exclusion are reported in Figure 1.

The final sample size was determined by the number of eligible patients enrolled during the study period. Because 46 definite PICC-CRBSI events occurred, the primary combined model was restricted to six prespecified predictor degrees of freedom, corresponding to approximately 7.7 events per predictor degree of freedom. Model complexity was constrained using the framework of Riley et al., accounting for the number of primary events, anticipated model fit, and a target global shrinkage factor ≥0.90 (Riley et al., 2020). Although this event count was modest, the model was not expanded beyond the prespecified six predictor degrees of freedom, and overfitting was addressed through continuous predictor modeling, avoidance of data-driven cut-point selection, L2 regularization, and patient-level bootstrap internal validation. All continuous biomarkers were retained in continuous form without data-driven cut-point selection, stepwise variable selection, unplanned interaction terms, or post-hoc expansion of model complexity. Variables with biological or clinical relevance that were not included in the primary combined model, including frailty, ICU/HDU care, baseline active infection, recent antimicrobial exposure, and active malignancy, were retained for descriptive comparison or prespecified sensitivity analyses rather than added to the primary model.

All PICC insertions were performed by vascular access nurses who had completed standardized institutional training and competency assessment. Power-injectable polyurethane PICCs were inserted into the basilic, brachial, or cephalic vein under ultrasound guidance using a modified Seldinger technique. A 4-Fr single-lumen catheter was preferred; a 5-Fr double-lumen catheter was used when simultaneous infusion of incompatible drugs was required. Catheter choice, insertion side, and lumen number were determined by clinical indication and vascular access assessment rather than study assignment.

Maximum sterile barrier precautions were implemented during insertion, including cap, mask, sterile gown, sterile gloves, and full-body draping. Skin was disinfected with 2% chlorhexidine gluconate in 70% isopropyl alcohol. The catheter tip was positioned at the lower superior vena cava or cavoatrial junction and confirmed by intracavitary electrocardiography or chest radiography before clinical use. The catheter was secured with a sutureless stabilization device and covered with a sterile transparent semipermeable dressing.

Needleless connectors were disinfected with 70% alcohol for at least 15 s and allowed to dry completely before each access. Transparent dressings were routinely scheduled for replacement every 7 days and were replaced earlier if wet, loose, contaminated, blood-saturated, non-occlusive, or otherwise disrupted. Daily nursing assessments documented catheter necessity, dressing integrity, puncture-site status, and signs of local or systemic infection when patients remained under inpatient care. For discharged patients, catheter maintenance information was abstracted from outpatient vascular access visits, infusion-center records, nursing notes, emergency visits, and readmission records. Because this was a retrospective study, catheter-care exposure was analyzed from documented routine-care records rather than from protocol-mandated prospective observation. Routine systemic antimicrobial prophylaxis and antimicrobial lock therapy were not administered solely for PICC infection prevention. All maintenance procedures were based on institutional practice aligned with international central-line infection-prevention guidance (O’Grady et al., 2011).

Systemic antimicrobial therapy given for non-catheter infections after PICC insertion was not treated as an exclusion criterion because it reflected routine clinical care in this population. However, antimicrobial exposure could reduce culture positivity, modify bloodstream infection incidence, and influence subsequent inflammatory status. Therefore, recent antimicrobial exposure before insertion and antimicrobial treatment during follow-up were extracted when available and considered in descriptive and sensitivity analyses. Antimicrobial therapy initiated after the 24-h landmark was not used as a baseline predictor to avoid immortal-time and post-landmark bias.

Follow-up started at the 24-h landmark and ended at the first of: definite PICC-CRBSI, all-cause death without prior infection, non-infectious PICC removal, loss of effective follow-up, or completion of the 60-day observation window. Patients lost to follow-up were censored on the date of the last valid clinical record confirming catheter and infection status. The primary time scale was days from the 24-h landmark, and catheter-days at risk accrued only while the PICC remained in place and before definite infection, death, non-infectious removal, loss of follow-up, or administrative censoring.

For patients undergoing PICC removal without confirmed infection at the time of the procedure, all microbiological records, clinical documentation, emergency department visits, and readmission data within 48 h after removal were reviewed to determine whether the episode met the prespecified PICC-CRBSI diagnostic criteria. If no qualifying infection was confirmed within the 48-h adjudication window, the removal was recorded as a non-infectious competing event on the actual removal date.

When definite PICC-CRBSI was diagnosed within 48 h after catheter removal, the episode was classified as a valid infection event. The event time was the collection time of the first qualifying diagnostic specimen; if the first specimen was collected after removal, the removal date was used as the analytical event time to avoid biased risk set classification. All-cause death without prior definite PICC-CRBSI was recorded as a competing event on the date of death.

For patients discharged with indwelling PICCs, follow-up incorporated outpatient vascular access records, infusion center records, emergency department visits, hospital readmissions, microbiology results, catheter maintenance logs, and standardized telephone follow-up when available. Outcome adjudication used all valid clinical data within the predefined observation window, irrespective of care setting. Follow-up completeness was assessed before database locking, and patients without a valid record confirming catheter status, infection status, removal, death, or completion of the 60-day window were censored at the last verifiable contact date.

### 2.3 Clinical variables, biomarkers, and data quality

All clinical variables were extracted using a prespecified case report form, including age, sex, body mass index, patient location at the 24-h landmark, PICC indication, active malignancy, diabetes mellitus, chronic kidney disease, dementia, recent systemic antibiotic exposure, total parenteral nutrition, ICU/HDU admission, baseline active infection, sepsis status, frailty level, and catheter-related variables. Active malignancy was defined as metastatic or recurrent malignancy, unremitted hematological malignancy, or malignancy diagnosed or treated within 6 months before PICC insertion. Recent antibiotic exposure was defined as systemic antibacterial treatment for ≥48 h within 14 days before PICC insertion. Baseline active infection was defined as clinically confirmed bacterial infection receiving systemic antibacterial treatment at the 24-h landmark, excluding primary bloodstream infection. Frailty was evaluated using the 9-point Clinical Frailty Scale based on functional status approximately 2 weeks before acute illness onset; a score ≥6 indicated moderate-to-severe frailty (Rockwood et al., 2005). For patients without a documented score, frailty was retrospectively assessed from pre-illness mobility, activities of daily living, cognitive function, and care dependency, without reference to subsequent catheter-related outcomes. Catheter-related variables included insertion vein, insertion side, lumen number, skin puncture attempts, catheter tip confirmation method, and early dressing disruption. Early dressing disruption was defined as a loose, wet, contaminated, blood-saturated, non-occlusive, or unexpectedly replaced dressing documented between PICC insertion and the 24-h landmark; post-landmark dressing abnormalities were not used as baseline predictors.

All laboratory values were those measured closest to PICC insertion within the preceding 24 h. Complete blood counts were performed using an XN-1000 hematology analyzer (Sysmex Corporation, Kobe, Japan). Serum C-reactive protein and albumin were measured using a cobas c702 chemistry analyzer, and procalcitonin was measured using the Elecsys BRAHMS Procalcitonin assay on a cobas e801 analyzer (Roche Diagnostics, Mannheim, Germany). Laboratory platforms, calibration records, reagent-lot documentation, internal quality-control results, and external quality-assessment records were reviewed during data cleaning. No major analytical-platform change affecting complete blood count, C-reactive protein, albumin, or procalcitonin measurement was identified during the study period.

The following indices were calculated from raw laboratory values:

$$NLR=\frac{absoluteneutrophilcount}{absolutelymphocytecount}$$

$$PLR=\frac{plateletcount}{absolutelymphocytecount}$$

$$SII=\frac{plateletcount\times absoluteneutrophilcount}{absolutelymphocytecount}$$

$$CAR=\frac{C-reactiveprotein\left(mg/L\right)}{serumalbumin\left(g/L\right)}$$

NLR and PLR were dimensionless; SII was expressed as ×10^9^/L; CAR was expressed as mg/g. SII, CAR, and procalcitonin were selected for the biomarker and combined models because they were routinely available within the 24-h landmark window and captured complementary immune-cell, platelet, inflammatory, bacterial-response, and nutritional dimensions. SII was prioritized over NLR and PLR to avoid redundant leukocyte-ratio entry under a modest event count. CAR was prioritized because it combines acute-phase inflammation with albumin-based nutritional reserve, which is clinically relevant in oldest-old PICC recipients. Procalcitonin was retained as a supplementary bacterial-inflammatory biomarker but was examined in sensitivity analyses because selective testing and missingness were expected. NLR and PLR were retained as exploratory biomarkers. IL-6, ferritin, and other cytokine markers were not included in the clinical prediction model because they were not routinely measured within the landmark window and would have introduced excessive missingness. These indices have been used in catheter-associated infection and infection-risk studies (Yang et al., 2022). Procalcitonin was analyzed separately as a supplementary biomarker (Wacker et al., 2013). Variable definitions, units, ascertainment windows, coding rules, analytical roles, and missingness are provided in Supplementary Table S1.

All baseline data were extracted by a trained investigator, and 10% of randomly selected records were independently re-extracted by a second investigator. Categorical agreement was assessed using percentage agreement and Cohen’s kappa; continuous agreement was assessed using the two-way random-effects single-measure absolute-agreement intraclass correlation coefficient. Discrepancies were classified as transcription errors, source-record ambiguity, timing-window errors, unit errors, or derived-variable calculation errors and were resolved by source-record review. Before database locking, range checks, unit checks, chronological validation, duplicate screening, and logical consistency checks were performed. Implausible values were corrected from source records or coded as missing, and all derived indices were recalculated centrally.

Missing data patterns were assessed before model fitting. Variables with >20% missingness were excluded from primary models; outcomes, event times, censoring times, and follow-up duration were not imputed. Variables with ≤20% missingness were imputed using multivariate imputation by chained equations with 20 datasets (van Buuren and Groothuis-Oudshoorn, 2011). Predictive mean matching was used for continuous variables, logistic regression for binary variables, and multinomial logistic regression for unordered categorical variables. Raw hematological and biochemical values were imputed before recalculation of NLR, PLR, SII, and CAR. Missingness for every candidate predictor and final-model predictor was reported in Supplementary Table S1. Patients with and without procalcitonin measurements were compared using standardized mean differences to assess selective testing bias. Sensitivity analyses included complete-case analysis, a model excluding procalcitonin, and delta-adjusted imputation of missing log_2_-transformed procalcitonin values.

### 2.4 Outcome definition and adjudication

The primary endpoint was definite PICC-CRBSI occurring within 60 days after the 24-h landmark. Qualifying clinical manifestations included new-onset fever, chills, rigor, hypotension, acute delirium, or unexplained systemic deterioration prompting bloodstream infection screening. Definite PICC-CRBSI required compatible symptoms plus at least one catheter-specific microbiological criterion: (i) the same microorganism with consistent antimicrobial susceptibility isolated from peripheral blood and catheter tip culture, with catheter tip growth ≥15 CFU by semiquantitative culture or ≥10^2^ CFU by quantitative culture; (ii) the same pathogen detected in paired catheter-drawn and peripheral blood cultures, with the catheter-drawn culture becoming positive at least 2 h earlier; or (iii) a catheter-to-peripheral quantitative blood-culture ratio ≥3:1. For differential time-to-positivity diagnosis, paired peripheral and catheter blood cultures collected within the same diagnostic episode were required. Blood cultures were collected before antimicrobial initiation or adjustment whenever clinically feasible.

A single positive blood culture for common skin commensals, including coagulase-negative staphylococci, was insufficient for definite diagnosis unless the same organism with a compatible susceptibility pattern was recovered from a second independently collected blood culture or catheter tip culture. Episodes with another established infection source were not attributed to the PICC without concurrent catheter-specific microbiological evidence. A qualifying infection identified within 48 h after PICC removal remained classified as catheter-related. The event date was the collection date of the first qualifying specimen; when the first qualifying specimen was obtained only after removal, the removal date was used as the analytical event time. Removal without a qualifying infection during the 48-h adjudication window was coded as non-infectious PICC removal on the actual removal date. These criteria were adapted from established guidance for intravascular catheter-related infection (Mermel et al., 2009).

Potential infection episodes were identified from positive blood or catheter tip cultures, paired catheter and peripheral blood cultures, catheter removal for suspected infection, antimicrobial escalation for suspected bloodstream infection, and clinical documentation of unexplained systemic deterioration. Routine microbiology personnel interpreted cultures and susceptibility results according to laboratory procedures and were not involved in prediction modeling or translational experiments. Endpoint adjudicators reviewed the diagnostic episode records independently and were blinded to predicted risk scores, risk strata, model coefficients, candidate biomarker roles, and translational results. Complete blinding to all clinical information was not feasible because symptoms, antimicrobial timing, catheter status, culture timing, and alternative infection sources were necessary for endpoint classification. However, adjudicators were not provided with the model-derived risk worksheet or the final predictor set during adjudication. Initial agreement was assessed using crude percentage agreement, Cohen’s kappa with 95% confidence interval, and separate agreement for definite infection versus non-definite episodes. Disagreements were categorized as microbiological confirmation disagreement, alternative-source disagreement, timing-window disagreement, or insufficient-documentation disagreement before resolution by consensus or a third senior infectious disease physician.

Probable PICC-related bloodstream infection was defined as compatible bloodstream infection occurring while the PICC was in place or within 48 h after removal, without a more plausible source but without sufficient catheter-specific evidence for definite diagnosis. Common skin commensals required at least two independently collected positive blood culture sets. Secondary outcomes were probable PICC-related bloodstream infection, all-cause bloodstream infection, catheter removal for suspected infection, and 60-day all-cause mortality. Definite infection incidence was expressed per 1,000 catheter-days with exact Poisson 95% confidence intervals. Catheter-days accrued from the landmark until definite infection, catheter removal, death, loss of follow-up, or day 60, whichever occurred first.

### 2.5 Model development, performance, and risk stratification

Three prognostic models were prespecified to separate the contribution of clinical and immune-inflammatory information. The clinical model included total parenteral nutrition, double-lumen PICC, multiple insertion attempts, early dressing disruption, Clinical Frailty Scale score ≥6, and ICU/HDU admission. The biomarker model included log_2_-transformed SII, CAR, and procalcitonin. The primary combined model comprised total parenteral nutrition, double-lumen PICC, early dressing disruption, log_2_(SII), log_2_(CAR), and log_2_(procalcitonin). This structure allowed comparison of catheter-related clinical prediction, biomarker-only prediction, and the incremental value of combining both domains while keeping the primary model limited to six predictor degrees of freedom. For CAR or procalcitonin values of zero, 0.01 was substituted before log_2_ transformation. Continuous predictors were standardized using the mean and standard deviation of their transformed values. During cross-validation and bootstrap resampling, all preprocessing parameters were estimated within each training or resampled dataset and applied to the corresponding validation partition. Final locked centering and scaling constants were estimated from the full cohort. Age was excluded from the primary model because the cohort was restricted to patients aged ≥80 years; continuous age was added in a prespecified sensitivity analysis.

All models were fitted using L2-penalized Fine–Gray proportional subdistribution hazards regression, with definite PICC-CRBSI designated as the event of interest and all-cause death without prior infection and non-infectious PICC removal treated as competing events (Fine and Gray, 1999). The 20 multiply imputed datasets were vertically stacked, and each imputed record received an analytical weight of 1/20. Ten-fold cross-validation was implemented at the patient level so that all imputed copies of the same patient remained within the same fold. The optimal penalty parameter λ was selected according to the one-standard-error rule applied to the cross-validated 60-day Brier score. The selected penalty was then applied to the weighted stacked dataset to estimate final regression coefficients and the baseline cumulative subdistribution hazard. The locked combined-model parameters and preprocessing constants are reported in Supplementary Table S2.

For a patient with predictor vector X, the linear predictor was calculated as:

$$LP=\beta_{1}X_{1}+\beta_{2}X_{2}+\cdots +\beta_{6}X_{6}$$

The predicted cumulative incidence of definite PICC-CRBSI by day 60 was calculated as:

$$\left.P_{60}=1-\exp\left\{-\right.H_{0}\left(60\right)\times \exp\left(LP\right)\right\}$$

Discrimination was assessed using the 60-day time-dependent area under the receiver operating characteristic curve (AUC), which quantified how well the model ranked patients who did and did not develop definite PICC-CRBSI by day 60. Calibration was examined using the calibration intercept, calibration slope, observed-to-expected ratio, flexible calibration curves, and Aalen–Johansen cumulative incidence estimates within predicted-risk strata. Calibration was interpreted as agreement between predicted absolute risk and observed competing-risk cumulative incidence, rather than as a test of statistical significance. Overall prediction error was quantified by the 60-day Brier score. Clinical utility was evaluated using decision-curve analysis across threshold probabilities from 2% to 30%; decision-curve analysis was interpreted as the net clinical benefit of using model-based risk classification compared with classifying all patients or no patients as high risk, without assuming that the model was ready for clinical implementation (Vickers and Elkin, 2006). The proportional subdistribution hazards assumption was evaluated using time-by-predictor interaction terms and graphical residual diagnostics. When evidence of non-proportionality was identified, a time-varying coefficient sensitivity model was fitted without altering the locked primary model.

IInternal validation used 1,000 patient-level bootstrap resamples. Each bootstrap iteration repeated the full analytical pipeline, including imputation, transformation, standardization, penalty selection, model fitting, and performance estimation. Optimism-corrected AUC, calibration slope, calibration intercept, observed-to-expected ratio, and Brier score were obtained by subtracting mean bootstrap optimism from apparent performance. Predicted 60-day risks were stratified as low (<5%), intermediate (5% to <15%), and high (≥15%). The thresholds of <5%, 5% to <15%, and ≥15% were therefore regarded as provisional interpretive categories rather than validated intervention triggers. These categories were used only for model interpretation, risk-group calibration, and prospective donor screening; they were not intended to trigger catheter removal, antimicrobial treatment, intensified surveillance, or berberine administration. Because external validation was absent, no public online calculator or clinical bedside score was released. The locked equation and worked example are provided only as a transparent implementation template for future validation studies.

Prespecified sensitivity analyses comprised restriction to patients aged ≥85 years, complete-case analysis, exclusion of baseline active infection, additional adjustment for active infection and sepsis, exclusion of common skin commensal infections without catheter tip confirmation, exclusion of deaths within 7 days after the landmark, inclusion of continuous age, omission of procalcitonin, cause-specific Cox regression, delta-adjusted imputation, and evaluation of discrimination and calibration across three calendar periods: 2019–2021, 2022–2023, and 2024–2025.

### 2.6 Plasma selection and berberine preparation

During the prospective translational phase, consecutive patients aged ≥80 years undergoing PICC placement were screened using the same eligibility criteria as the clinical cohort. Peripheral blood was collected before PICC insertion into EDTA-anticoagulated and lithium-heparin tubes. Lithium-heparin blood was transported on ice and centrifuged at 1,500 × g for 15 min at 4 °C within 60 min. Plasma was aliquoted into 250-µL single-use portions, stored at −80 °C, and subjected to at most one freeze–thaw cycle.

The locked combined and biomarker models were applied at the 24-h landmark without refitting. Low-inflammatory-risk donors had a combined-model risk <5% and biomarker-model risk ≤4.1%; high-inflammatory-risk donors had a combined-model risk ≥15% and biomarker-model risk ≥13.8%. Both criteria were required only to enrich low- and high-inflammatory plasma conditions for the *in vitro* experiment; donor strata were not used to guide clinical care. Twelve donors were enrolled per group and frequency-matched by sex and 5-year age category. Donors were excluded for active infection, positive blood culture, sepsis at collection, systemic corticosteroid exposure >10 mg/day prednisone equivalent, biological immunosuppressive therapy within 4 weeks, or visibly hemolyzed or lipemic plasma. Samples were tested individually, and laboratory personnel remained blinded to donor group until analysis completion.

Berberine chloride (purity ≥98%; Sigma-Aldrich, B3251) was dissolved in cell-culture-grade dimethyl sulfoxide to prepare a 20-mM stock solution, filtered through a 0.22-µm membrane, aliquoted, protected from light, and stored at −20 °C. Working solutions were freshly prepared before each experiment, and all berberine-treated and vehicle-control conditions contained 0.1% dimethyl sulfoxide. The berberine concentrations were selected for exploratory *in vitro* exposure and were not assumed to represent clinically achievable systemic concentrations in patients. Materials, strain-specific exposure concentrations, antimicrobial susceptibility profiles, experimental conditions, quality controls, and analysis units are summarized in Supplementary Table S3.

### 2.7 PICC-coupon biofilm experiment

The biofilm experiment used *Staphylococcus* epidermidis ATCC 35984 and six nonduplicate clinical S. epidermidis isolates recovered from definite PICC-CRBSI episodes. This species was selected because coagulase-negative staphylococci were common in the clinical cohort and because S. epidermidis is a standard catheter-biofilm organism. The experiment was a focused Gram-positive biofilm model and did not represent Gram-negative or fungal PICC-CRBSI. Clinical isolates were identified using a MALDI Biotyper Sirius system (Bruker Daltonics), stored at −80 °C in tryptic soy broth with 20% glycerol, subcultured twice before testing, and summarized by susceptibility profile in Supplementary Table S3.

Overnight cultures were grown in tryptic soy broth at 37 °C with agitation at 180 rpm and adjusted to approximately 1 × 10^6^ CFU/mL. Berberine MICs were determined by broth microdilution in cation-adjusted Mueller–Hinton broth according to CLSI M07, with *Staphylococcus aureus* ATCC 29213 as the quality-control strain (Clinical and Laboratory Standards Institute, 2024). Each strain was exposed to vehicle, 1/8 MIC, 1/4 MIC, or 1/2 MIC berberine to assess subinhibitory biofilm modulation (Zhao et al., 2023). MIC values and exposure concentrations are listed in Supplementary Table S3.

Unused sterile polyurethane PICC material from the same catheter family used clinically was cut into 10-mm segments and placed in 24-well plates. Each well received 1 mL of bacterial suspension in tryptic soy broth with 1% glucose and the assigned berberine concentration, followed by static incubation at 37 °C for 24 h. Vehicle-treated cultures, sterile coupons, and concentration-matched berberine blanks served as growth, background, and optical controls.

Separate coupons were used for viable-count and crystal-violet assays. For viable biofilm burden, coupons were washed three times with phosphate-buffered saline, transferred to 1 mL phosphate-buffered saline, sonicated for 5 min at 40 kHz, vortexed for 60 s, serially diluted, and plated on tryptic soy agar. Colonies were counted after 24 h at 37 °C and expressed as log_10_ CFU/coupon. For biomass, matched coupons were fixed with methanol, stained with 0.1% crystal violet, destained with 30% acetic acid, and measured at 590 nm. Planktonic growth was measured at 600 nm after berberine-blank subtraction. Background-corrected OD600 values were used for inference; vehicle-normalized values were used only for presentation.

Each strain–dose–endpoint condition was evaluated in three technical wells across three independent experimental runs. Technical wells were averaged before analysis, yielding one strain–dose–run value as the analysis unit. Representative SEM or confocal images were included only when image acquisition and fixation quality were adequate. Imaging was descriptive and was not used as a primary quantitative endpoint.

For sterile biofilm-conditioned medium, untreated 24-h ATCC 35984 biofilms on PICC coupons were washed and incubated in serum-free RPMI-1640 for 4 h at 37 °C. Conditioned medium was centrifuged at 3,000 × g for 10 min, filtered through a 0.22-µm membrane, confirmed sterile by 48-h aerobic culture, aliquoted, and stored at −80 °C. Three independent batches were prepared for macrophage experiments.

### 2.8 Plasma-conditioned macrophage experiment

THP-1 human monocytic cells were maintained in RPMI-1640 medium supplemented with 10% heat-inactivated fetal bovine serum, 1% penicillin–streptomycin, and 50 µM 2-mercaptoethanol at 37 °C with 5% CO_2_. Cell identity was confirmed by short-tandem-repeat profiling, and *mycoplasma* testing was negative before experiments. Cells between passages 4 and 15 were seeded at 2 × 10^5^ cells/well, differentiated with 100 ng/mL phorbol 12-myristate 13-acetate for 48 h, and rested for 24 h. Antibiotics were omitted during experimental exposure.

Differentiated macrophage-like cells were pretreated for 1 h with vehicle, 5 µM berberine, or 10 µM berberine, then exposed to serum-free RPMI-1640 containing 10% individual donor plasma and 10% sterile biofilm-conditioned medium. Each donor was tested under all three berberine conditions on the same plate, and plasma samples were not pooled. Individual donor plasma samples were treated as biological replicates, whereas duplicate wells were technical replicates. One of three independently prepared conditioned-medium batches was assigned to each plate, with low- and high-risk donors balanced across plates. Plate positions were randomized, personnel remained blinded to donor group, and all conditions contained 0.1% dimethyl sulfoxide. Berberine concentrations were used for exploratory *in vitro* inflammatory modulation and cytotoxicity screening based on prior macrophage NF-κB studies (Gong et al., 2019).

Cells were harvested at 6 h for NF-κB pathway analysis, and culture supernatants and viability measurements were collected at 24 h. IL-6 was the primary inflammatory endpoint. TNF-α, phospho-NF-κB p65 Ser536/total p65 ratio, and cell viability were prespecified secondary endpoints. IL-1β, IL-8, MCP-1, and other cytokines were not prespecified endpoints and were not included in the primary inferential analysis unless available as exploratory measurements. IL-6 and TNF-α were measured in duplicate using Quantikine ELISA kits. Duplicate coefficient of variation ≤15%, spike recovery of 80%–120%, and acceptable dilution parallelism were required for assay acceptance.

For Western blotting, cells were lysed in RIPA buffer with protease/phosphatase inhibitors. Equal protein amounts were resolved by SDS-PAGE, transferred to PVDF membranes, and probed for phospho-p65, total p65, and β-actin. Phospho-p65 was normalized to total p65, total p65 was checked against β-actin, and values were expressed relative to a common reference lysate on each gel. Saturated exposures were repeated, and uncropped membrane images were retained.

Cell viability was assessed using Cell Counting Kit-8 after subtraction of cell-free background absorbance. Background-corrected absorbance values were used for inference, and donor-normalized percentages were used for presentation. A cytokine reduction was interpreted as occurring without substantial cytotoxicity only when mean donor-normalized viability remained ≥85%.

### 2.9 Statistical analysis

Continuous clinical variables were summarized as mean ± standard deviation or median with interquartile range, and categorical variables as counts and percentages. Baseline characteristics were compared descriptively between patients with and without definite PICC-CRBSI using absolute standardized mean differences; univariable hypothesis testing was not used for predictor selection. The 60-day cumulative incidence of definite PICC-CRBSI was estimated using the Aalen–Johansen estimator with death and non-infectious PICC removal treated as competing events. Crude event counts, crude proportions, and competing-risk cumulative incidence estimates were reported separately. Incidence density was expressed per 1,000 catheter-days with exact Poisson 95% confidence intervals. Prediction-model development, internal validation, calibration, decision-curve analysis, and sensitivity analyses followed the framework in Section 2.5.

For the PICC-coupon biofilm experiment, the mean of three technical wells within each strain–dose–run condition was the analysis unit. Log_10_ CFU/coupon was analyzed using a linear mixed-effects model with MIC-normalized berberine exposure as a fixed categorical effect, bacterial strain as a random intercept, and experimental run as a fixed blocking factor. A linear trend test used MIC fractions of 0, 0.125, 0.250, and 0.500. Pairwise comparisons against vehicle used estimated marginal means with Holm adjustment. Crystal violet OD590, background-corrected planktonic OD600, and biomass-to-growth ratio were analyzed as secondary endpoints using analogous models. Principal biofilm findings were reported as adjusted mean differences with 95% confidence intervals, in addition to adjusted P values.

For the macrophage experiment, duplicate wells were averaged to produce one donor–dose observation. Log-transformed IL-6 was analyzed using a mixed-effects model with inflammatory risk group, berberine dose, and risk-group-by-dose interaction as fixed effects; donor as a random intercept; and plate as a fixed blocking factor. The global berberine dose effect on IL-6 was the primary experimental test. The interaction term assessed whether the dose response differed between low- and high-risk plasma conditions. TNF-α, phospho-p65/total-p65 ratio, and viability were analyzed using the same repeated-measures framework. Back-transformed cytokine results were reported as geometric mean ratios with 95% confidence intervals; p65 and viability results were reported as adjusted mean differences with 95% confidence intervals.

All statistical tests were two-sided, and adjusted P < 0.05 was considered statistically significant. No missing laboratory endpoint values were imputed. Observations were excluded only for prespecified assay failure, contamination, or failure to meet quality-control criteria. Clinical analyses were performed in R 4.4.2 using mice, riskRegression, cmprsk, and pec, with the prespecified penalized Fine–Gray implementation. Laboratory mixed-effects models used lme4 and emmeans. Complete scripts, package versions, transformation steps, model parameters, and fixed random-number seeds were archived before final analysis.

## 3 Results

### 3.1 Cohort characteristics and clinical outcomes

A total of 386 patients aged 80–97 years entered the 24-h landmark cohort; 213 (55.2%) were women. During 19,528 catheter-days at risk, 46 patients developed definite PICC-CRBSI, corresponding to an incidence density of 2.36 per 1,000 catheter-days (95% CI, 1.72–3.14). Median time from the landmark to infection was 17 days (IQR, 14–23), and median time from PICC insertion to infection was 18 days. Twenty-one patients died without prior definite infection, 212 underwent non-infectious PICC removal, and 107 completed 60 days without a primary or competing event; no patient was lost to follow-up. Probable PICC-related bloodstream infection occurred in 11 patients, all-cause bloodstream infection in 68, and catheter removal for suspected infection in 34.

Among the 46 definite PICC-CRBSI episodes, Gram-positive bacteria accounted for 29 infections (63.0%), Gram-negative bacteria for 12 (26.1%), and fungi for 5 (10.9%). Coagulase-negative staphylococci were the most frequent organisms, accounting for 16 infections (34.8%), followed by *Staphylococcus aureus* and Enterobacterales, with 8 infections each (17.4% each), *Enterococcus* species and *Candida* species, with 5 infections each (10.9% each), and other Gram-negative bacteria, with 4 infections (8.7%).

Procalcitonin had the highest missingness among primary-model variables at 15.8% (61/386), followed by the Clinical Frailty Scale at 6.0%, C-reactive protein at 4.1%, lymphocyte count and early dressing disruption at 2.1% each, and albumin at 1.8%. No primary-model variable exceeded the 20% missingness threshold. Patients without procalcitonin measurements were more frequently free of baseline active infection and less frequently managed in ICU/HDU than those with procalcitonin measurements, with absolute standardized mean differences of 0.31 and 0.28, respectively; all other absolute standardized mean differences were <0.25. Systemic antimicrobial therapy after the 24-h landmark was recorded in 219 patients (56.7%), reflecting routine treatment of non-catheter infections and other clinical indications. Post-landmark antimicrobial therapy was not included as a baseline predictor.

Duplicate extraction of 39 randomly selected records yielded a Cohen’s kappa of 0.93 (95% CI, 0.89–0.97) for categorical variables and an intraclass correlation coefficient of 0.987 (95% CI, 0.981–0.992) for continuous variables. Among 67 potential bloodstream infection episodes independently reviewed, the two adjudicators initially agreed on 62 episodes (92.5%; Cohen’s kappa, 0.84; 95% CI, 0.72–0.95). Initial agreement was 43/46 (93.5%) for definite infection episodes and 19/21 (90.5%) for non-definite or alternative-source episodes. The five initial disagreements involved alternative-source attribution in 2 episodes, catheter-specific microbiological confirmation in 2 episodes, and timing-window classification in 1 episode. All disagreements were resolved by consensus or third-reviewer adjudication before database locking.

Compared with patients without definite infection, patients with definite PICC-CRBSI were descriptively older and more frequently had active malignancy, moderate-to-severe frailty, total parenteral nutrition, double-lumen PICC, and early dressing disruption. They also had lower lymphocyte counts and albumin, and higher white blood cell count, C-reactive protein, NLR, SII, and CAR (Table 1).

**TABLE 1:** Baseline characteristics according to 60-day definite PICC-related bloodstream infection status.

Characteristic	No definite PICC-CRBSI n=340	Definite PICC-CRBSI n=46	Absolute SMD
Age, years	84.5 (82.0–87.0)	88.0 (85.0–90.0)	0.733
Female	188/340 (55.3%)	25/46 (54.3%)	0.019
Body mass index, kg/m^2^	21.6 (19.3–24.0)	20.0 (18.0–22.2)	0.431
Active malignancy	156/340 (45.9%)	32/46 (69.6%)	0.494
Diabetes mellitus	110/340 (32.4%)	12/46 (26.1%)	0.138
Chronic kidney disease	113/340 (33.2%)	17/46 (37.0%)	0.078
Clinical frailty scale	5.0 (4.0–5.0)	5.5 (4.3–6.0)	0.465
Clinical frailty scale ≥6	79/321 (24.6%)	21/42 (50.0%)	0.544
Recent antibiotic exposure	160/340 (47.1%)	24/46 (52.2%)	0.102
Total parenteral nutrition	150/340 (44.1%)	28/46 (60.9%)	0.340
ICU/HDU care	87/340 (25.6%)	14/46 (30.4%)	0.108
Double-lumen PICC	107/340 (31.5%)	28/46 (60.9%)	0.617
Insertion attempts ≥2	65/340 (19.1%)	10/46 (21.7%)	0.065
Early dressing disruption	44/332 (13.3%)	10/46 (21.7%)	0.225
White blood cell count, ×10^9^/L	7.31 (5.91–8.47)	8.53 (7.04–9.43)	0.581
Lymphocyte count, ×10^9^/L	1.29 (0.98–1.66)	0.96 (0.58–1.20)	0.883
C-reactive protein, mg/L	16.81 (9.02–31.55)	31.26 (13.42–60.19)	0.600
Albumin, g/L	34.41 (31.20–36.67)	31.74 (29.57–35.30)	0.616
Procalcitonin, ng/mL	0.099 (0.048–0.187)	0.146 (0.070–0.273)	0.216
NLR	3.70 (2.42–5.90)	6.00 (4.23–9.74)	0.611
SII, ×10^9^/L	815.7 (477.0–1,324.4)	1,332.5 (904.2–2,155.0)	0.639
CAR, mg/g	0.46 (0.22–0.94)	0.98 (0.35–1.95)	0.540

Data are median (IQR) or n/available n (%). Baseline summaries are based on observed data before multiple imputation. SMD, standardized mean difference; PICC, peripherally inserted central catheter; CRBSI, catheter-related bloodstream infection; ICU, intensive care unit; HDU, high-dependency unit; NLR, neutrophil-to-lymphocyte ratio; SII, systemic immune-inflammation index; CAR, C-reactive protein-to-albumin ratio.

### 3.2 Model performance and risk stratification

The clinical and biomarker models achieved apparent 60-day time-dependent AUCs of 0.724 (95% CI, 0.642–0.803) and 0.717 (95% CI, 0.636–0.797), respectively. The combined model yielded an apparent AUC of 0.758 (95% CI, 0.682–0.834), Brier score of 0.0885, calibration intercept of 0.04 (95% CI, −0.60–0.69), calibration slope of 1.03 (95% CI, 0.69–1.36), and observed-to-expected ratio of 0.98. After 1,000 patient-level bootstrap repetitions, the optimism-corrected AUC was 0.731, Brier score was 0.0920, calibration intercept was 0.02, calibration slope was 0.91, and observed-to-expected ratio was 1.02. No material violation of the proportional subdistribution hazards assumption was detected, with a global time-interaction P value of 0.41 and no individual predictor P value <0.10.

The locked combined model classified 123 patients as low risk, 171 as intermediate risk, and 92 as high risk. Crude infection proportions were 3.3% (4/123), 8.8% (15/171), and 29.3% (27/92), respectively. Corresponding 60-day Aalen–Johansen cumulative incidences were 3.2% (95% CI, 1.0%–7.4%), 8.5% (95% CI, 4.9%–13.3%), and 28.1% (95% CI, 19.3%–37.5%). Mean predicted risks were 3.0%, 9.1%, and 28.9%, respectively. The calibration curve showed close agreement between mean predicted risk and observed competing-risk cumulative incidence across the three risk strata. In decision-curve analysis, the combined model showed greater net benefit than classifying all or no patients as high risk across threshold probabilities of 5%–25%, and greater net benefit than the single-component models across most thresholds between 7% and 22%. Because the model was not externally validated, decision-curve results were interpreted as internal evidence of potential utility rather than evidence for immediate clinical implementation.

**FIGURE 2:**
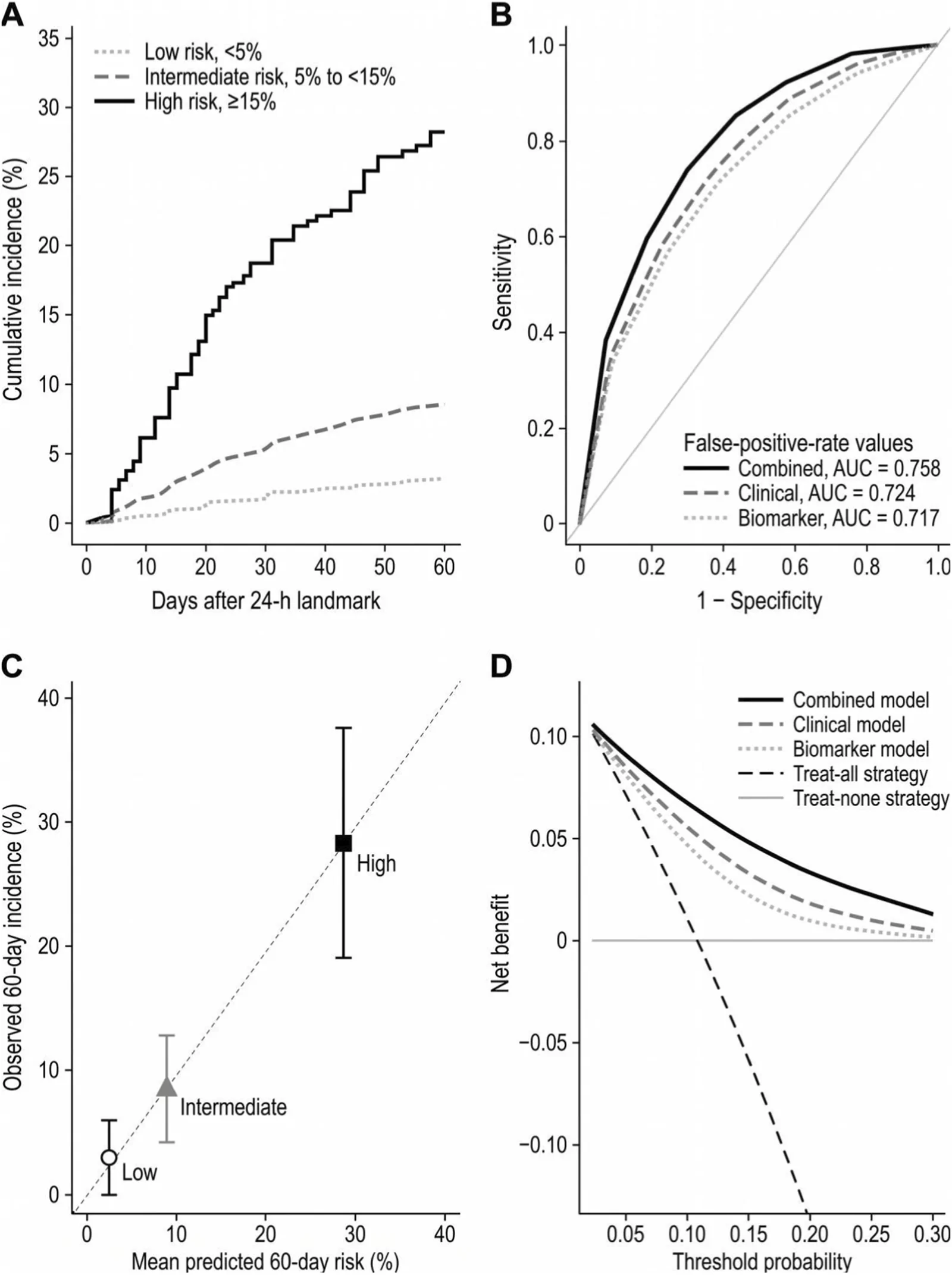
Performance of the combined competing-risk model. **(A)** Aalen–Johansen cumulative incidence curves of definite PICC-related bloodstream infection stratified according to the three predicted-risk categories. **(B)** 60-day time-dependent receiver operating characteristic curves for the clinical model, biomarker model, and primary combined model. **(C)** Observed Aalen–Johansen cumulative incidence estimates plotted against mean model-predicted 60-day risk. **(D)** Decision-curve analysis comparing the net clinical benefit of the three prediction models. In all analyses, all-cause death without prior infection and non-infectious PICC removal were treated as competing events.

The ordered risk gradient was preserved in prespecified sensitivity analyses, including complete-case analysis, restriction to patients aged ≥85 years, exclusion or adjustment for baseline infection, exclusion of early deaths, exclusion of skin-commensal infections without catheter tip confirmation, inclusion of continuous age, omission of procalcitonin, cause-specific Cox regression, and delta-adjusted imputation. Calendar-period analyses for 2019–2021, 2022–2023, and 2024–2025 did not show directional deterioration in discrimination or calibration (Table 2). Model discrimination, calibration, risk-stratified cumulative incidence, and decision-curve results are shown in Figure 2.

**TABLE 2:** Prediction-model performance, risk stratification, and sensitivity analyses.

Model	Day-60 AUC (95% CI)	Brier score	Calibration intercept	Calibration slope	Observed/Expected
A. Model performance					
Clinical model	0.724 (0.642–0.803)	0.0968	0.08	0.91	1.07
Biomarker model	0.717 (0.636–0.797)	0.0943	0.06	0.94	1.04
Combined model, apparent	0.758 (0.682–0.834)	0.0885	0.04 (−0.60–0.69)	1.03 (0.69–1.36)	0.98
Combined model, bootstrap corrected	0.731	0.0920	0.02	0.91	1.02

Risk stratum	Patients, n	Definite PICC-CRBSI, n	Crude event proportion	Day-60 Aalen–Johansen incidence (95% CI)	Mean predicted risk
B. Risk-stratified outcomes					
Low, <5%	123	4	3.3%	3.2% (1.0%–7.4%)	3.0%
Intermediate, 5% to <15%	171	15	8.8%	8.5% (4.9%–13.3%)	9.1%
High, ≥15%	92	27	29.3%	28.1% (19.3%–37.5%)	28.9%

Analysis	Patients, n	Events, n	Day-60 AUC	Brier score
C. Prespecified sensitivity analyses				
Complete-case cohort	304	41	0.758	0.0973
Patients aged ≥85 years	208	38	0.726	0.1270
Combined model excluding procalcitonin	386	46	0.756	0.0913
Exclusion of baseline active infection	247	25	0.744	0.0785
Adjustment for baseline infection and sepsis	386	46	0.754	0.0891
Exclusion of deaths within 7 days	379	46	0.756	0.0876
Exclusion of skin-commensal events without catheter-tip confirmation	386	40	0.749	0.0806
Combined model including continuous age	386	46	0.766	0.0869
Cause-specific cox model	386	46	0.748	0.0895

Calendar period	Patients, n	Events, n	Day-60 AUC	Observed/expected
D. Calendar-period stability				
2019–2021	118	13	0.721	0.94
2022–2023	126	14	0.736	1.02
2024–2025	142	19	0.729	1.05

AUC, time-dependent area under the receiver operating characteristic curve; PCT, procalcitonin.

Across the four prespecified delta-adjusted procalcitonin imputation scenarios, the optimism-corrected AUC of the combined model ranged from 0.728 to 0.734, and the Brier score ranged from 0.0917 to 0.0925.

### 3.3 Berberine reduced PICC-coupon biofilm formation

Berberine MICs for the 7 S. epidermidis strains ranged from 64 to 256 μg/mL, and all strains formed measurable biofilms on polyurethane PICC coupons. Mean viable biofilm burden decreased from 7.656 ± 0.202 log_10_ CFU/coupon under vehicle exposure to 7.494 ± 0.207, 7.266 ± 0.251, and 6.919 ± 0.260 log_10_ CFU/coupon at 1/8, 1/4, and 1/2 MIC, respectively. Adjusted differences from vehicle were −0.162 (95% CI, −0.240 to −0.084), −0.390 (95% CI, −0.468 to −0.311), and −0.737 (95% CI, −0.815 to −0.659) log_10_ CFU/coupon, respectively, with Holm-adjusted P < 0.001 for each comparison.

**FIGURE 3:**
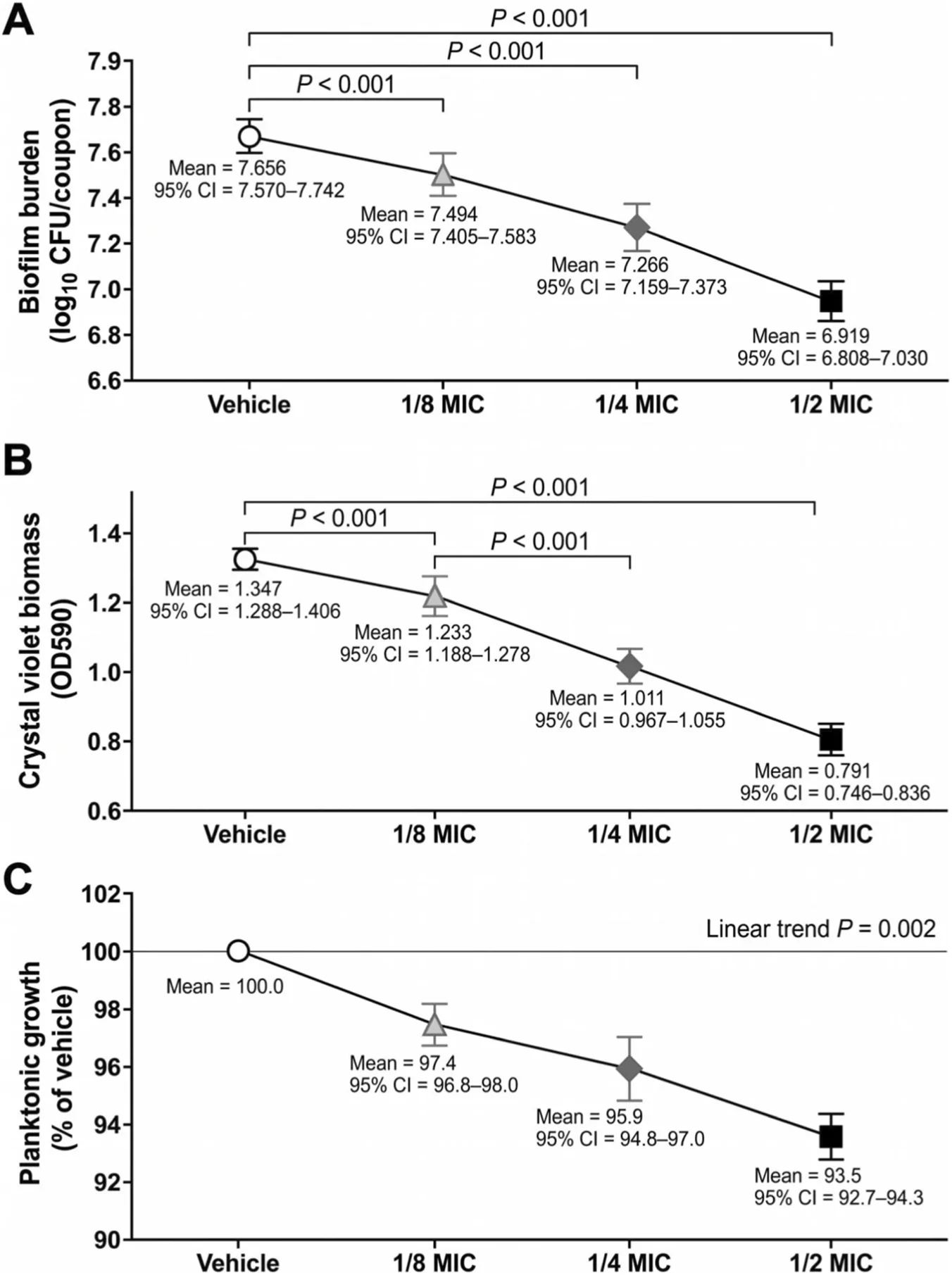
Effect of subinhibitory berberine exposure on PICC-coupon biofilm. **(A)** Viable biofilm burden expressed as log_10_ CFU per coupon. **(B)** Crystal violet biofilm biomass expressed as OD_590_. **(C)** Planktonic bacterial growth expressed as percentage of the vehicle control condition. Individual data points represent strain–run mean values, and horizontal markers with error bars indicate estimated marginal means with their associated 95% confidence intervals. Vehicle control, 1/8 MIC, 1/4 MIC, and 1/2 MIC conditions are represented by open circles, gray triangles, dark-gray diamonds, and filled black squares, respectively.

Crystal violet biomass decreased in parallel, with adjusted OD590 differences from vehicle of −0.114 (95% CI, −0.160 to −0.068), −0.336 (95% CI, −0.382 to −0.290), and −0.556 (95% CI, −0.602 to −0.510) at 1/8, 1/4, and 1/2 MIC, respectively. The prespecified linear trend was significant for both viable burden and biomass (P < 0.001 for both). Planktonic growth declined more modestly, remaining at 93.5% of vehicle at 1/2 MIC; the linear trend for background-corrected OD600 was significant (P = 0.002). The biomass-to-growth ratio decreased across berberine concentrations (P < 0.001). Representative SEM or confocal images showed reduced surface-associated biofilm density and less continuous biofilm coverage at 1/2 MIC compared with vehicle exposure. These images were used as qualitative support and were not included in statistical inference. Strain–run distributions are shown in Figure 3, and numerical results are summarized in Table 3.

**TABLE 3:** Effects of subinhibitory berberine exposure on PICC-coupon biofilm.

Berberine exposure	log_10_ CFU/coupon, mean ± SD	Adjusted difference vs. vehicle (95% CI)	Crystal violet OD590, mean ± SD	Adjusted OD590 difference vs. vehicle (95% CI)	Planktonic growth relative to vehicle
Vehicle	7.656 ± 0.202	Reference	1.347 ± 0.139	Reference	100.0%
1/8 MIC	7.494 ± 0.207	−0.162 (−0.240 to −0.084)	1.233 ± 0.105	−0.114 (−0.160 to −0.068)	97.4% ± 1.4%
1/4 MIC	7.266 ± 0.251	−0.390 (−0.468 to −0.311)	1.011 ± 0.103	−0.336 (−0.382 to −0.290)	95.9% ± 2.6%
1/2 MIC	6.919 ± 0.260	−0.737 (−0.815 to −0.659)	0.791 ± 0.105	−0.556 (−0.602 to −0.510)	93.5% ± 1.9%

Technical well replicates were averaged within each strain–dose–run condition to yield a single independent observation for analysis. For both viable biofilm burden and crystal violet biomass endpoints, all pairwise comparisons of each berberine concentration against the vehicle control condition yielded Holm-adjusted P values below 0.001. Statistical inference for planktonic growth was based on raw background-corrected OD_600_ values; percentage values relative to the vehicle control are presented for descriptive purposes. MIC, minimum inhibitory concentration; CFU, colony-forming units.

### 3.4 Berberine attenuated plasma-conditioned macrophage responses

The macrophage experiment included 12 low-inflammatory-risk and 12 high-inflammatory-risk donors. Each group included 7 women; median age was 86 years (IQR, 83–89) in the low-risk group and 87 years (IQR, 84–90) in the high-risk group. Mean combined-model predicted risk was 3.1% ± 0.8% and 24.6% ± 7.2%, respectively. Individual donor plasma samples served as biological replicates, and duplicate wells were averaged before analysis. All cytokine duplicates met the prespecified coefficient-of-variation threshold, with a maximum duplicate CV of 12.4%. Spike recovery ranged from 91.2% to 108.6%, dilution parallelism was acceptable, and no observation was excluded for assay failure.

Under vehicle conditions, IL-6 was higher with high-risk than low-risk plasma (616.3 ± 53.6 vs. 402.9 ± 62.2 pg/mL; adjusted geometric mean ratio, 1.55; 95% CI, 1.38–1.74; P < 0.001). Across both risk groups, berberine reduced IL-6 in a concentration-dependent pattern. Compared with vehicle, 5 µM berberine reduced IL-6 by 9.5% (adjusted geometric mean ratio, 0.905; 95% CI, 0.884–0.927), and 10 µM reduced IL-6 by 23.4% (adjusted geometric mean ratio, 0.766; 95% CI, 0.742–0.790), with Holm-adjusted P < 0.001 for both comparisons. The global dose effect was significant (P < 0.001), whereas the risk-group-by-dose interaction was not significant (P = 0.976).

**FIGURE 4:**
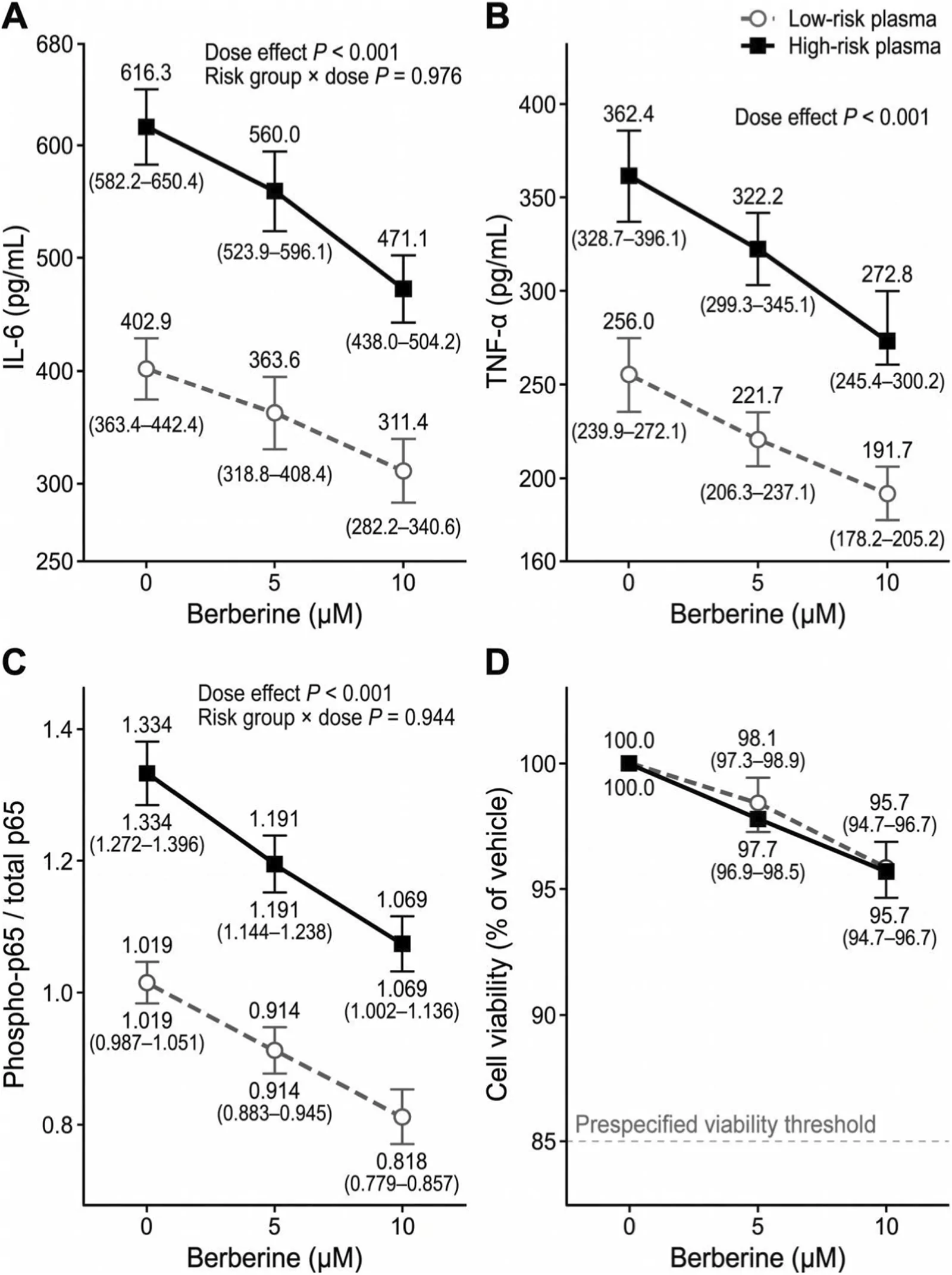
Plasma-conditioned macrophage responses to berberine. **(A)** Interleukin-6 (IL-6) concentration in culture supernatants. **(B)** Tumor necrosis factor-α (TNF-α) concentration in culture supernatants. **(C)** Ratio of phosphorylated NF-κB p65 (Ser536) to total p65 protein. **(D)** Cell viability expressed relative to the corresponding donor-specific vehicle control condition. Individual data points represent donor-level observations; lines and error bars indicate estimated marginal means with their associated 95% confidence intervals. Low-inflammatory-risk plasma conditions are represented by open circles and dashed lines, and high-inflammatory-risk plasma conditions by filled squares and solid lines.

The prespecified mechanistic endpoint showed parallel reductions in the phospho-p65/total-p65 ratio. Adjusted ratios versus vehicle were 0.894 (95% CI, 0.875–0.913) at 5 μM and 0.802 (95% CI, 0.782–0.823) at 10 μM, with Holm-adjusted P < 0.001 for both comparisons. The risk-group-by-dose interaction for phospho-p65/total-p65 was not significant (P = 0.944). TNF-α also decreased across berberine concentrations in both plasma-risk groups (global dose effect P < 0.001). Exploratory cytokine measurements, when available, showed the same directional pattern: IL-1β decreased by 14.8%, IL-8 by 18.6%, and MCP-1 by 16.2% at 10 µM berberine compared with vehicle. These exploratory mediators were not included in the primary inferential hierarchy. Donor-normalized mean viability ranged from 95.7% to 100.0%, and no condition crossed the prespecified 85% cytotoxicity threshold. Numerical results are summarized in Table 4 and shown in Figure 4.

**TABLE 4:** Plasma-conditioned macrophage responses according to risk group and berberine concentration.

Plasma-risk group	Berberine	IL-6 (pg/mL)	TNF-α (pg/mL)	Phospho-p65/total-p65	Cell viability relative to donor vehicle
Low risk	0 µM	402.9 ± 62.2	256.0 ± 25.4	1.019 ± 0.050	100.0%
Low risk	5 µM	363.6 ± 70.5	221.7 ± 24.3	0.914 ± 0.049	98.1% ± 1.3%
Low risk	10 µM	311.4 ± 46.0	191.7 ± 21.3	0.818 ± 0.061	95.7% ± 1.5%
High risk	0 µM	616.3 ± 53.6	362.4 ± 53.0	1.334 ± 0.097	100.0%
High risk	5 µM	560.0 ± 56.8	322.2 ± 36.0	1.191 ± 0.074	97.7% ± 1.3%
High risk	10 µM	471.1 ± 52.1	272.8 ± 43.1	1.069 ± 0.105	95.7% ± 1.5%

Data are mean ± standard deviation. Values represent donor-level observations after duplicate technical wells were averaged. Cell viability was normalized to the corresponding donor-specific vehicle condition, defined as 100%; formal inference for viability used background-corrected absorbance values. Exploratory IL-1β, IL-8, and MCP-1 results are not shown in the table and should be replaced with final locked values if retained. IL-6, interleukin-6; TNF-α, tumor necrosis factor-α; NF-κB, nuclear factor kappa B.

## 4 Discussion

This single-center study combined competing-risk prognostic modeling with an exploratory translational substudy in patients aged ≥80 years undergoing PICC placement. Three findings emerged. First, definite PICC-related bloodstream infection occurred in 46 of 386 patients, and affected patients showed greater frailty, higher catheter-related exposure, lymphopenia, systemic inflammation, and lower albumin. Second, the combined model integrating catheter-related variables with immune-inflammatory biomarkers showed moderate internally validated discrimination and preserved an ordered gradient of 60-day infection risk after accounting for death and non-infectious catheter removal. Third, subinhibitory berberine reduced S. epidermidis biofilm burden on PICC coupons and attenuated cytokine and phospho-p65 responses in plasma-conditioned macrophages without reducing viability below the prespecified cytotoxicity threshold. These findings should be interpreted as internally consistent clinical and experimental observations, not as evidence that the model is ready for clinical deployment or that berberine is clinically effective for PICC infection prevention.

PICC-related bloodstream infection reflects the combined effects of host vulnerability, treatment intensity, catheter characteristics, microbiological exposure, and maintenance conditions. Prior studies have associated PICC bloodstream infection with intensive care exposure, prolonged dwell time, parenteral nutrition, and multilumen catheters (Herc et al., 2017). Recent prediction-model work has shown that routinely available clinical variables can support early infection-risk estimation, but existing models vary substantially in outcome definitions, predictor selection, validation design, and target populations (Sakai et al., 2023; Zeng et al., 2025). Most published models were developed in broader adult, oncology, neonatal, or mixed central-line populations rather than in oldest-old PICC recipients. The present model differs by using a fixed 24-h prediction landmark, restricting the primary model to six prespecified predictor degrees of freedom, retaining continuous biomarker information, and treating death and non-infectious catheter removal as competing events. This framework is clinically relevant in very advanced-age cohorts, where death or catheter removal may occur before infection can be observed. However, the bootstrap-corrected AUC remained moderate, so the model is better viewed as a candidate risk-stratification tool for future validation than as a definitive bedside decision rule.

The immune-inflammatory profile associated with infection risk was consistent with advanced-age vulnerability. Immunosenescence, inflammaging, frailty, malnutrition, and multimorbidity may weaken host defense and alter the clinical expression of infection (Theodorakis et al., 2024; Calder et al., 2022). SII summarizes neutrophil, lymphocyte, and platelet components, whereas CAR combines acute-phase inflammation with albumin-associated nutritional reserve. Their contribution to the combined model probably reflects a broad host-reserve phenotype rather than a catheter-specific infection signal. Procalcitonin contributed limited additional discrimination, and the model excluding procalcitonin performed similarly. This finding is important because procalcitonin was the most frequently missing predictor and may have been ordered selectively in patients with stronger clinical suspicion of infection. SII and CAR may therefore be more consistently implementable in routine datasets, but none of these biomarkers should be interpreted as diagnostic evidence of PICC-CRBSI without compatible symptoms and catheter-specific microbiological confirmation.

The three-tier risk stratification may help describe baseline risk heterogeneity, but the thresholds remain provisional. The high-risk group contained most definite infections and may justify closer review of catheter necessity, lumen requirements, dressing integrity, connector access practice, infusion exposure, and early systemic deterioration in future implementation studies. A low predicted risk did not eliminate infection, and standard catheter-care practices remain necessary in all patients. The decision-curve findings indicate potential internal net benefit across selected threshold ranges, but they do not justify immediate clinical use because the model has not undergone external validation. For this reason, no online calculator, nomogram, or simplified bedside score should be promoted for clinical decision-making at this stage. The locked equation may be useful for independent validation studies, but catheter removal, antimicrobial therapy, or intensified surveillance should not be triggered solely by the current model.

The berberine experiments provide a mechanistic extension focused on the biofilm–inflammation axis, but the translational gap remains substantial. S. epidermidis was selected because coagulase-negative staphylococci were common in the clinical cohort and because S. epidermidis is a canonical catheter-biofilm organism. This choice made the coupon experiment biologically focused, but it did not represent the full microbiological spectrum of PICC-CRBSI, including Gram-negative bacteria and fungi. Prior studies have reported that berberine can affect staphylococcal biofilm formation and macrophage inflammatory signaling through pathways involving NF-κB and related metabolic-inflammatory regulators (Ma et al., 2020; Zhang et al., 2017). In this study, viable biofilm burden and biomass decreased more clearly than planktonic growth, and macrophage IL-6, TNF-α, and phospho-p65 responses decreased without major cytotoxicity. These results support further study of berberine as a candidate antibiofilm and immunomodulatory compound *in vitro*, but the concentrations used here should not be assumed to be clinically achievable through systemic administration in oldest-old patients. The study also did not test a berberine-coated PICC, antimicrobial lock formulation, sustained-release device surface, animal catheter model, or clinical prevention strategy.

Several limitations should be emphasized. The clinical cohort was retrospective and single-center, leaving potential selection bias, documentation errors, residual confounding, local practice effects, and incomplete capture of post-discharge events. Frailty sometimes required retrospective reconstruction from records, and procalcitonin was not measured uniformly. Multiple imputation, L2 penalization, and bootstrap correction reduced but did not eliminate overfitting or bias. The 46 primary events constrained model complexity, risk-stratum precision, and assessment of interactions or temporal instability. Despite standardized adjudication, PICC-CRBSI may have been missed when paired blood cultures or catheter tip cultures were unavailable, and classification of common skin commensals remained challenging. The translational substudy was also limited by the small number of bacterial strains and donors, static coupon biofilms, one reference strain for conditioned medium, THP-1-derived macrophage-like cells, and short-term exposure conditions. The experimental system did not reproduce catheter flow, repeated intraluminal drug exposure, protein conditioning layers, host immune-cell diversity, systemic pharmacokinetics, or toxicity risks in very old adults. Future work should include prospective multicenter external validation, calibration updating across institutions, comparison with simplified bedside scores, and translational testing in flow-based biofilm systems, animal catheter models, and eventually safety-focused studies of berberine-coated PICCs or catheter-lock approaches.

## 5 Conclusion

In patients aged ≥80 years undergoing PICC placement, a single-center competing-risk model combining total parenteral nutrition, double-lumen PICC, early dressing disruption, SII, CAR, and procalcitonin provided preliminary internal stratification of 60-day definite PICC-related bloodstream infection risk while accounting for death and non-infectious catheter removal. The model should not be used as a clinical decision tool until externally validated in independent multicenter cohorts. In a separate exploratory substudy, subinhibitory berberine reduced S. epidermidis biofilm burden on polyurethane PICC material and attenuated plasma-conditioned macrophage inflammatory responses *in vitro* without substantial cytotoxicity. These laboratory findings support further investigation of the biofilm–inflammation pathway but do not establish berberine as a clinical therapy, catheter coating, or antimicrobial lock intervention.

## Data Availability

The original contributions presented in the study are included in the article/Supplementary Material, further inquiries can be directed to the corresponding authors.

## References

[B1] Asokan S. Banerjee N. Saleem M. Atiyah H. M. Pandey R. K. Abbas R. K. (2026a). Healthcare associated infections (HAI): insights into epidemiology, microbiology, and diagnostics. Diagn. Microbiol. Infect. Dis. 115, 117376. 10.1016/j.diagmicrobio.2026.117376 41865593

[B2] Asokan S. Choudekar A. Jagadeesan A. Rudresh S. M. Ali A. Napte S. U. (2026b). Molecular diagnostics in clinical microbiology: advances, applications, and future directions. Diagn. Microbiol. Infect. Dis. 114, 117223. 10.1016/j.diagmicrobio.2025.117223 41406849

[B3] Asokan S. Pandey R. K. Jalil M. A. Abd Alhussen S. K. Yousif S. I. Abbas R. K. (2026c). Biofilm associated infections on medical devices: pathogenesis, diagnostic challenges, and control strategies. Microbe 11, 100712. 10.1016/j.microb.2026.100712

[B4] Calder P. C. Carr A. C. Gombart A. F. Eggersdorfer M. (2022). Nutrition, immunosenescence, and infectious disease. Nutrients 14, 4016. 10.3390/nu14194016 36235669 PMC9572163

[B5] Cao L. Tao Y. Lei S. He S. Peng Y. Liu S. (2025). Models for predicting the risk of bloodstream infections associated with peripherally inserted central venous catheters: a scoping review. PLoS One 20, e0333466. 10.1371/journal.pone.0333466 41052038 PMC12500127

[B6] Clinical and Laboratory Standards Institute (2024). Methods for Dilution Antimicrobial Susceptibility Tests for Bacteria that Grow Aerobically. 12th ed. Wayne, PA: Clinical and Laboratory Standards Institute. CLSI standard M07.

[B7] Collins G. S. Reitsma J. B. Altman D. G. Moons K. G. M. (2015). Transparent reporting of a multivariable prediction model for individual prognosis or diagnosis (TRIPOD): the TRIPOD statement. Ann. Intern. Med. 162, 55–63. 10.7326/M14-0697 25560714

[B8] Fine J. P. Gray R. J. (1999). A proportional hazards model for the subdistribution of a competing risk. J. Am. Stat. Assoc. 94, 496–509. 10.1080/01621459.1999.10474144

[B9] Fulop T. Larbi A. Dupuis G. Le Page A. Frost E. H. Cohen A. A. (2018). Immunosenescence and inflamm-aging as two sides of the same coin: friends or foes? Front. Immunol. 8, 1960. 10.3389/fimmu.2017.01960 29375577 PMC5767595

[B10] Gong J. Li J. Dong H. Chen G. Qin X. Hu M. (2019). Inhibitory effects of berberine on proinflammatory M1 macrophage polarization through interfering with the interaction between TLR4 and MyD88. BMC Complement. Altern. Med. 19, 314. 10.1186/s12906-019-2710-6 31744490 PMC6862859

[B11] Grau D. Clarivet B. Lotthé A. Bommart S. Parer S. (2017). Complications with peripherally inserted central catheters used in hospitalized patients and outpatients: a prospective cohort study. Antimicrob. Resist Infect. Control 6, 18. 10.1186/s13756-016-0161-0 28149507 PMC5273851

[B12] Herc E. Patel P. Washer L. L. Conlon A. Flanders S. A. Chopra V. (2017). A model to predict central-line-associated bloodstream infection among patients with peripherally inserted central catheters: the MPC score. Infect. Control Hosp. Epidemiol. 38, 1155–1166. 10.1017/ice.2017.167 28807074

[B13] Lafuente C. E. Terradas R. R. Civit C. A. Sardelli D. G. López C. H. Formatger D. G. (2013). Risk factors of catheter-associated bloodstream infection: systematic review and meta-analysis. PLoS One 18, e0282290. 10.1371/journal.pone.0282290 PMC10035840 36952393

[B14] Lee J. H. Kim E. T. Shim D. J. Kim I. J. Byeon J. H. Lee I. J. (2019). Prevalence and predictors of peripherally inserted central catheter-associated bloodstream infections in adults: a multicenter cohort study. PLoS One 14, e0213555. 10.1371/journal.pone.0213555 30845210 PMC6405206

[B15] Ma J. Wang Y. Yang J. Yang M. Chang K. A. Zhang L. (2020). Berberine inhibits pro-inflammatory cytokine-induced IL-6 and CCL11 production *via* modulation of STAT6 pathway in human bronchial epithelial cells. Int. J. Med. Sci. 17, 1464–1473. 10.7150/ijms.44899 32624703 PMC7330667

[B16] Mermel L. A. Allon M. Bouza E. Craven D. E. Flynn P. O’Grady N. P. (2009). Clinical practice guidelines for the diagnosis and management of intravascular catheter-related infection: 2009 update by the infectious diseases society of America. Clin. Infect. Dis. 49, 1–45. 10.1086/599376 19489710 PMC4039170

[B17] Mo C. Wang L. Zhang J. Numazawa S. Tang H. Tang X. (2014). The crosstalk between Nrf2 and AMPK signal pathways is important for the anti-inflammatory effect of berberine in LPS-stimulated macrophages and endotoxin-shocked mice. Antioxid. Redox. Signal. 20, 574–588. 10.1089/ars.2012.5116 23875776 PMC3901384

[B18] O’Grady N. P. Alexander M. Burns L. A. Dellinger E. P. Garland J. Heard S. O. (2011). Guidelines for the prevention of intravascular catheter-related infections. Clin. Infect. Dis. 52, e162–e193. 10.1093/cid/cir257 21460264 PMC3106269

[B19] Riley R. D. Ensor J. Snell K. I. E. Harrell F. E. J. Martin G. P. Reitsma J. B. (2020). Calculating the sample size required for developing a clinical prediction model. BMJ 368, m441. 10.1136/bmj.m441 32188600

[B20] Rockwood K. Song X. MacKnight C. Bergman H. Hogan D. B. McDowell I. (2005). A global clinical measure of fitness and frailty in elderly people. CMAJ 173, 489–495. 10.1503/cmaj.050051 16129869 PMC1188185

[B21] Sadighi Akha A. A. (2018). Aging and the immune system: an overview. J. Immunol. Methods 463, 21–26. 10.1016/j.jim.2018.08.005 30114401

[B22] Safdar N. Maki D. G. (2005). Risk of catheter-related bloodstream infection with peripherally inserted central venous catheters used in hospitalized patients. Chest 128, 489–495. 10.1378/chest.128.2.489 16100130

[B23] Sakai H. Iwata M. Terasawa T. (2023). External validation of the Michigan PICC catheter-associated bloodstream infections score (MPC score) for predicting the risk of peripherally inserted central catheter-associated bloodstream infections: a single-center study in Japan. Infect. Control Hosp. Epidemiol. 44, 480–483. 10.1017/ice.2021.497 34924068

[B24] Theodorakis N. Chatzigeorgiou A. Chavakis T. Kreouzi M. Kalantzi S. Spyridaki A. (2024). Immunosenescence: how aging increases susceptibility to bacterial infections and virulence factors. Microorganisms 12, 2052. 10.3390/microorganisms12102052 39458361 PMC11510421

[B25] van Buuren S. Groothuis-Oudshoorn K. (2011). Mice: multivariate imputation by chained equations in R. J. Stat. Softw. 45, 1–67. 10.18637/jss.v045.i03

[B26] Vickers A. J. Elkin E. B. (2006). Decision curve analysis: a novel method for evaluating prediction models. Med. Decis. Mak. 26, 565–574. 10.1177/0272989X06295361 17099194 PMC2577036

[B27] von Elm E. Altman D. G. Egger M. Pocock S. J. Gøtzsche P. C. Vandenbroucke J. P. (2007). The strengthening the reporting of Observational studies in epidemiology (STROBE) statement: guidelines for reporting observational studies. PLoS Med. 4, e296. 10.1371/journal.pmed.0040296 17941714 PMC2020495

[B28] Wacker C. Prkno A. Brunkhorst F. M. Schlattmann P. (2013). Procalcitonin as a diagnostic marker for sepsis: a systematic review and meta-analysis. Lancet Infect. Dis. 13, 426–435. 10.1016/S1473-3099(12)70323-7 23375419

[B29] Wang X. Yao X. Zhu Z. A. Tang T. Dai K. Sadovskaya I. (2009). Effect of berberine on Staphylococcus epidermidis biofilm formation. Int. J. Antimicrob. Agents 34, 60–66. 10.1016/j.ijantimicag.2008.10.033 19157797

[B30] Yang J. Wang H. Hua Q. Wu J. Wang Y. (2022). Diagnostic value of systemic inflammatory response index for catheter-related bloodstream infection in patients undergoing haemodialysis. J. Immunol. Res. 2022, 7453354–7453359. 10.1155/2022/7453354 35132381 PMC8817844

[B31] Zeng F. Li W. Ye H. Xie C. Zhong H. (2025). A predictive model for catheter-related bloodstream infection in neonates with peripherally inserted central catheter. Front. Med. 12, 1665068. 10.3389/fmed.2025.1665068 41195176 PMC12583055

[B32] Zhang H. Shan Y. Wu Y. Xu C. Yu X. Zhao J. (2017). Berberine suppresses LPS-induced inflammation through modulating Sirt1/NF-κB signaling pathway in RAW264.7 cells. Int. Immunopharmacol. 52, 93–100. 10.1016/j.intimp.2017.08.032 28888780

[B33] Zhao N. Isguven S. Evans R. Schaer T. P. Hickok N. J. (2023). Berberine disrupts staphylococcal proton motive force to cause potent anti-staphylococcal effects *in vitro* . Biofilm 5, 100117. 10.1016/j.bioflm.2023.100117 37090161 PMC10113750

[B34] Zhao Z. Yuan S. Zhang X. Zhang X. Li H. Liu X. (2025). Neutrophil-to-lymphocyte ratio and CRP-to-albumin ratio in the prediction of catheter-related bloodstream infection among maintenance hemodialysis patients: a synergistical optimization algorithm. Front. Med. (Lausanne) 12, 1612057. 10.3389/fmed.2025.1612057 40978747 PMC12443806

