# Supplementary material for: Immune-inflammatory prediction of PICC-related bloodstream infection in oldest-old adults with exploratory berberine biofilm–inflammation experiments
Source: Front Pharmacol. 2026 Aug 18;17:1914929. doi: 10.3389/fphar.2026.1914929 (PMC13529987; doi:10.3389/fphar.2026.1914929)
Supplement: Supplementary file 1 [file Table1.docx]

Supplementary Table S1. Definitions, coding rules, ascertainment windows, analytical roles, and missingness of study variables

A. Patient and clinical variables

| **Variable** | **Definition and coding** | **Unit/category** | **Ascertainment window** | **Analytical role** | **Missingness, n (%)** |
| --- | --- | --- | --- | --- | --- |
| Patient identifier | Unique de-identified code assigned to the first eligible PICC episode per patient | Alphanumeric | Cohort entry | Record linkage | 0 (0.0%) |
| Age | Age on the insertion date | Years, continuous | PICC insertion | Descriptive variable; sensitivity-model predictor | 0 (0.0%) |
| Age ≥85 years | Age of at least 85 years | 0 = no; 1 = yes | PICC insertion | Subgroup analysis | 0 (0.0%) |
| Sex | Biological sex recorded in the medical record | Female/male | Index admission | Descriptive variable; donor matching | 0 (0.0%) |
| Body mass index | Weight divided by height squared | kg/m² | Closest reliable value within 7 days before insertion | Descriptive and auxiliary variable | 19 (4.9%) |
| Clinical location | Patient location at the 24-h landmark | General ward, oncology ward, surgical ward, ICU/HDU, or other | 24-h landmark | Descriptive variable | 2 (0.5%) |
| PICC indication | Principal reason for PICC placement | Antimicrobial therapy, chemotherapy, parenteral nutrition, difficult access, or other | PICC insertion | Descriptive variable | 0 (0.0%) |
| Active malignancy | Metastatic or recurrent cancer, hematological malignancy not in remission, or cancer diagnosed or treated within 6 months before PICC insertion | 0 = no; 1 = yes | PICC insertion | Confounding assessment; sensitivity analysis | 3 (0.8%) |
| Diabetes mellitus | Documented diabetes or current glucose-lowering treatment | 0 = no; 1 = yes | Before or at PICC insertion | Descriptive variable | 1 (0.3%) |
| Chronic kidney disease | Documented chronic kidney disease or persistent eGFR <60 mL/min/1.73 m² | 0 = no; 1 = yes | Before PICC insertion | Descriptive variable | 4 (1.0%) |
| Dementia | Documented dementia before the index admission | 0 = no; 1 = yes | Before admission | Descriptive and auxiliary variable | 5 (1.3%) |
| Recent antibiotic exposure | Systemic antibacterial treatment for ≥48 h during the 14 days before PICC insertion | 0 = no; 1 = yes | Days −14 to 0 | Descriptive variable; auxiliary variable; sensitivity analysis | 6 (1.6%) |
| Baseline active infection | Clinically treated bacterial infection at the 24-h landmark, excluding primary bloodstream infection | 0 = no; 1 = yes | 24-h landmark | Descriptive variable; sensitivity analysis | 4 (1.0%) |
| Baseline infection site | Principal site of active infection | Respiratory, urinary, abdominal, skin/soft tissue, bone/joint, other, or none | 24-h landmark | Descriptive and subgroup analysis | 7 (1.8%) |
| Sepsis at landmark | Sepsis attributed to a documented non-bloodstream source | 0 = no; 1 = yes | 24-h landmark | Sensitivity-model covariate | 5 (1.3%) |
| Clinical Frailty Scale | Usual functional state approximately 2 weeks before acute illness onset | Score 1–9 | Pre-illness baseline | Source variable | 23 (6.0%) |
| Moderate-to-severe frailty | Clinical Frailty Scale score ≥6 | 0 = no; 1 = yes | Pre-illness baseline | Clinical-model predictor; descriptive comparison | 23 (6.0%) |
| Total parenteral nutrition | Initiated or prescribed by the 24-h landmark for an intended duration of ≥48 h | 0 = no; 1 = yes | PICC insertion to 24-h landmark | Clinical- and combined-model predictor | 0 (0.0%) |
| ICU/HDU care | ICU or high-dependency unit care before or at the 24-h landmark | 0 = no; 1 = yes | PICC insertion to 24-h landmark | Clinical-model predictor; sensitivity-model covariate | 2 (0.5%) |

B. Catheter-related variables

| **Variable** | **Definition and coding** | **Unit/category** | **Ascertainment window** | **Analytical role** | **Missingness, n (%)** |
| --- | --- | --- | --- | --- | --- |
| Insertion vein | Vein used for PICC placement | Basilic, brachial, or cephalic | PICC insertion | Descriptive variable | 2 (0.5%) |
| Insertion side | Arm used for PICC placement | Left/right | PICC insertion | Descriptive variable | 0 (0.0%) |
| Number of lumens | Number of functional catheter lumens | 1 or 2 | PICC insertion | Source variable | 0 (0.0%) |
| Double-lumen PICC | Catheter with two functional lumens | 0 = no; 1 = yes | PICC insertion | Clinical- and combined-model predictor | 0 (0.0%) |
| Number of skin punctures | Total number of skin punctures during the insertion procedure | Integer | PICC insertion | Source variable | 5 (1.3%) |
| Multiple insertion attempts | Two or more skin punctures during a single insertion procedure | 0 = no; 1 = yes | PICC insertion | Clinical-model predictor | 5 (1.3%) |
| Tip-confirmation method | Method used to confirm catheter-tip position | Intracavitary electrocardiography or chest radiography | Before catheter use | Descriptive and quality-control variable | 1 (0.3%) |
| Early dressing disruption | Loose, wet, contaminated, blood-saturated, edge-curling, non-occlusive, or unexpectedly replaced dressing | 0 = no; 1 = yes | PICC insertion to 24-h landmark | Clinical- and combined-model predictor | 8 (2.1%) |

C. Laboratory variables and derived immune-inflammatory indices

| **Variable** | **Definition and Coding** | **Unit/Category** | **Ascertainment Window** | **Analytical Role** | **Missingness, n (%)** |
| --- | --- | --- | --- | --- | --- |
| White blood cell count | Total peripheral leukocyte count | ×10⁹/L | Closest value within 24 h before PICC insertion | Descriptive biomarker | 7 (1.8%) |
| Absolute neutrophil count | Absolute peripheral neutrophil count | ×10⁹/L | Closest value within 24 h before PICC insertion | Component of NLR and SII | 8 (2.1%) |
| Absolute lymphocyte count | Absolute peripheral lymphocyte count | ×10⁹/L | Closest value within 24 h before PICC insertion | Component of NLR, PLR, and SII | 8 (2.1%) |
| Platelet count | Peripheral platelet count | ×10⁹/L | Closest value within 24 h before PICC insertion | Component of PLR and SII | 7 (1.8%) |
| C-reactive protein | Serum C-reactive protein | mg/L | Closest value within 24 h before PICC insertion | Component of CAR | 16 (4.1%) |
| Serum albumin | Serum albumin | g/L | Closest value within 24 h before PICC insertion | Component of CAR | 7 (1.8%) |
| Procalcitonin | Serum procalcitonin | ng/mL | Closest value within 24 h before PICC insertion | Biomarker- and combined-model predictor; sensitivity analysis | 61 (15.8%) |
| NLR | Absolute neutrophil count divided by absolute lymphocyte count | Dimensionless | Derived from pre-insertion values | Exploratory biomarker | 9 (2.3%) |
| PLR | Platelet count divided by absolute lymphocyte count | Dimensionless | Derived from pre-insertion values | Exploratory biomarker | 9 (2.3%) |
| SII | Platelet count × absolute neutrophil count ÷ absolute lymphocyte count | ×10⁹/L | Derived from pre-insertion values | Biomarker- and combined-model predictor | 10 (2.6%) |
| CAR | C-reactive protein in mg/L divided by serum albumin in g/L | mg/g | Derived from pre-insertion values | Biomarker- and combined-model predictor | 18 (4.7%) |

D. Outcomes, follow-up variables, and model outputs

| **Variable** | **Definition and coding** | **Unit/category** | **Ascertainment window** | **Analytical role** | **Missingness, n (%)** |
| --- | --- | --- | --- | --- | --- |
| Definite PICC-CRBSI | Compatible clinical syndrome with catheter-specific microbiological confirmation | Event code 1 | Landmark through day 60, including qualifying diagnoses within 48 h after catheter removal | Primary outcome | 0 (0.0%) |
| Probable PICC-related bloodstream infection | Compatible bloodstream infection without another likely source but insufficient catheter-specific microbiological evidence | Secondary event | Landmark through day 60 | Secondary outcome | 0 (0.0%) |
| All-cause bloodstream infection | Any adjudicated bloodstream infection regardless of source | Secondary event | Landmark through day 60 | Secondary outcome | 0 (0.0%) |
| Removal for suspected infection | PICC removal prompted wholly or partly by suspected catheter infection | Secondary event | Landmark through day 60 | Secondary outcome | 0 (0.0%) |
| Death without prior definite PICC-CRBSI | Death before the primary event | Event code 2 | Landmark through day 60 | Competing event | 0 (0.0%) |
| Non-infectious PICC removal | Catheter removal for a non-infectious reason without qualifying infection during the 48-h adjudication window | Event code 3 | Landmark through day 60 | Competing event recorded on removal date | 0 (0.0%) |
| Event-free completion | No primary or competing event by day 60 | Event code 0 | Landmark through day 60 | Administrative censoring | 0 (0.0%) |
| Loss of follow-up | Last date on which catheter and infection status were reliably known | Event code 0 | Landmark through day 60 | Censoring | 0 (0.0%) |
| Follow-up time | Time from the 24-h landmark to primary event, competing event, censoring, or day 60 | Days | Landmark through day 60 | Time-to-event scale | 0 (0.0%) |
| Catheter-days at risk | Time with the PICC in place after the 24-h landmark and before infection, death, removal, loss of follow-up, or day 60 | Catheter-days | Landmark through day 60 | Incidence-density denominator | 0 (0.0%) |
| Calendar period | Period of PICC insertion | 2019–2021, 2022–2023, or 2024–2025 | PICC insertion | Temporal-stability analysis | 0 (0.0%) |
| Predicted 60-day risk | Cumulative incidence estimated by the locked combined model | Probability, 0–1 | Calculated at the 24-h landmark | Individual prediction | Not applicable |
| Risk stratum | Category based on predicted 60-day risk | Low <5%; intermediate 5% to <15%; high ≥15% | Calculated at the 24-h landmark | Exploratory interpretation and prospective donor screening; not a treatment threshold | Not applicable |

Notes: PICC, peripherally inserted central catheter; ICU, intensive care unit; HDU, high-dependency unit; NLR, neutrophil-to-lymphocyte ratio; PLR, platelet-to-lymphocyte ratio; SII, systemic immune-inflammation index; CAR, C-reactive protein-to-albumin ratio; CRBSI, catheter-related bloodstream infection. Missingness was calculated in the final 24-hour landmark cohort of 386 patients. Raw hematological and biochemical measurements were imputed before derived immune-inflammatory indices were recalculated. Variables with >20% missingness were excluded from the primary models. Outcomes, competing-event indicators, event dates, censoring dates, follow-up time, and catheter-days at risk were not imputed.
