# Supplementary material for: Immune-inflammatory prediction of PICC-related bloodstream infection in oldest-old adults with exploratory berberine biofilm–inflammation experiments
Source: Front Pharmacol. 2026 Aug 18;17:1914929. doi: 10.3389/fphar.2026.1914929 (PMC13529987; doi:10.3389/fphar.2026.1914929)
Supplement: Supplementary file 2 [file Table2.docx]

**Supplementary Table S2.** Prediction-model structure, locked parameters, validation procedures, and sensitivity analyses

A. Prespecified model structure

| **Model** | **Predictors** | **Predictor form** | **Purpose** |
| --- | --- | --- | --- |
| Clinical model | Total parenteral nutrition; double-lumen PICC; multiple insertion attempts; early dressing disruption; Clinical Frailty Scale ≥6; ICU/HDU care | Six binary indicators | Clinical and catheter-related prediction |
| Biomarker model | SII; CAR; procalcitonin | Three log₂-transformed and standardized continuous variables | Immune-inflammatory prediction |
| Combined model | Total parenteral nutrition; double-lumen PICC; early dressing disruption; SII; CAR; procalcitonin | Three binary and three log₂-transformed standardized variables | Primary 60-day prediction model |

B. Predictor preprocessing and locked combined-model parameters

| **Predictor** | **Coding or transformation** | **Centering mean** | **Scaling SD** | **Penalized coefficient** |
| --- | --- | --- | --- | --- |
| Total parenteral nutrition | 0 = no; 1 = yes | Not centered | Not scaled | 0.310 |
| Double-lumen PICC | 0 = no; 1 = yes | Not centered | Not scaled | 0.620 |
| Early dressing disruption | 0 = no; 1 = yes | Not centered | Not scaled | 0.270 |
| SII | log₂(SII) | 9.740 | 1.050 | 0.350 |
| CAR | log₂(CAR); replace zero with 0.01 | −1.050 | 1.440 | 0.240 |
| Procalcitonin | log₂(PCT); replace zero with 0.01 | −3.210 | 1.370 | 0.100 |

The final cross-validated L2 penalty parameter was:

$$\lambda=0.087$$

The locked baseline cumulative subdistribution hazard at day 60 was:

$$H_{0}(60)=0.0795$$

Raw neutrophil, lymphocyte, platelet, C-reactive protein, albumin, and procalcitonin values were imputed before passive recalculation of derived indices. The identical locked transformations, centering values, scaling constants, coefficients, and baseline cumulative subdistribution hazard were applied to prospective plasma donors without model refitting.

C. Individual risk calculation

Standardized biomarker values were calculated as:

$$Z_{SII}=\frac{\log_{2} (SII)-9.740}{1.050}$$

$$Z_{CAR}=\frac{\log_{2} (CAR^{*})-(-1.050)}{1.440}$$

$$Z_{PCT}=\frac{\log_{2} (PCT^{*})-(-3.210)}{1.370}$$

where CAR*=0.01 if the observed CAR value equaled zero and otherwise equaled the observed CAR value, and PCT*=0.01 if the observed procalcitonin value equaled zero and otherwise equaled the observed procalcitonin value.

The locked linear predictor was:

$$LP=0.310(TPN)+0.620(double-lumenPICC)+0.270(earlydressingdisruption)+0.350Z_{SII}+0.240Z_{CAR}+0.100Z_{PCT}$$

All binary predictors were coded as 0 for absence and 1 for presence. The predicted 60-day cumulative incidence of definite PICC-CRBSI was calculated as:

$$P_{60}=1-exp\{-0.0795\times exp(LP)\}$$

Worked example. For an illustrative patient receiving total parenteral nutrition, with a double-lumen PICC in situ, without early dressing disruption, with an SII of 1,200 ×10⁹/L, a CAR of 0.80 mg/g, and a procalcitonin concentration of 0.15 ng/mL:

$$Z_{SII}=0.466,\quad Z_{CAR}=0.506,\quad Z_{PCT}=0.345$$

$$LP=1.249$$

$$P_{60}=23.9\%$$

Based on the calculated predicted risk, this patient would be assigned to the high-risk stratum.

D. Model fitting and internal validation

| **Aspect** | **Prespecified Procedure** |
| --- | --- |
| Multiple imputation | Twenty datasets generated using chained equations |
| Derived biomarkers | Raw component measurements imputed before passive recalculation |
| Dataset combination | Vertical stacking with each imputed record weighted 1/20 |
| Cross-validation | Patient-level 10-fold cross-validation |
| Penalty selection | One-standard-error rule based on the 60-day Brier score |
| Primary model | L2-penalized Fine–Gray regression |
| Internal validation | 1,000 patient-level bootstrap samples |
| Bootstrap pathway | Repeated imputation, preprocessing, penalty selection, fitting, and performance estimation |
| Discrimination | Day-60 time-dependent AUC |
| Calibration | Intercept, slope, observed-to-expected ratio, flexible curve, and risk-group calibration |
| Prediction error | Day-60 Brier score |
| Clinical utility | Decision-curve analysis at thresholds of 2%–30% |
| Proportionality | Time-by-predictor interactions and graphical residual assessment |
| Temporal stability | Evaluation across three calendar periods |

E. Risk categories

| **Predicted 60-day cumulative incidence** | **Risk category** | **Interpretation** |
| --- | --- | --- |
| <5% | Low | Lower predicted infection risk |
| 5% to <15% | Intermediate | Moderate predicted infection risk |
| ≥15% | High | Higher predicted infection risk |

The prespecified risk categories were used exclusively for model interpretation, risk-group calibration, and prospective plasma donor screening. They did not independently mandate catheter removal, antimicrobial treatment, intensified surveillance, or berberine administration.

F. Prespecified sensitivity analyses

| **Analysis** | **Purpose** |
| --- | --- |
| Patients aged ≥85 years | Assess robustness in the oldest subgroup |
| Complete-case cohort | Assess dependence on multiple imputation |
| Exclusion of baseline active infection | Reduce influence of pre-existing infection |
| Adjustment for active infection and sepsis | Examine residual inflammation-related confounding |
| Model excluding procalcitonin | Assess informative testing and implementation without PCT |
| Exclusion of deaths within 7 days | Examine influence of early competing mortality |
| Exclusion of incompletely confirmed skin-commensal events | Increase microbiological specificity |
| Inclusion of continuous age | Assess residual age-related prediction |
| Cause-specific Cox model | Compare cause-specific and subdistribution approaches |
| Delta-adjusted imputation | Examine departures from the missing-at-random assumption |
| Calendar-period analysis | Assess temporal changes in incidence, discrimination, and calibration |
