# Supplementary material for: Immune-inflammatory prediction of PICC-related bloodstream infection in oldest-old adults with exploratory berberine biofilm–inflammation experiments
Source: Front Pharmacol. 2026 Aug 18;17:1914929. doi: 10.3389/fphar.2026.1914929 (PMC13529987; doi:10.3389/fphar.2026.1914929)
Supplement: Supplementary file 3 [file Table3.docx]

**Supplementary Table S3.** Materials, experimental conditions, quality controls, and analysis units

A. Principal materials and instruments

| **Category** | **Material** | **Specification** | **Manufacturer/source** | **Catalog number/model** | **Use** |
| --- | --- | --- | --- | --- | --- |
| Clinical catheter material | Power-injectable polyurethane PICC | Unused sterile 10-mm segments from the same catheter family used clinically | BD/Bard Access Systems | PowerPICC SOLO2; 4-Fr single-lumen and 5-Fr dual-lumen models | Coupon biofilm model |
| Reference strain | Staphylococcus epidermidis RP62A | Biofilm-producing reference strain | ATCC | 35984 | Reference strain |
| Clinical strains | Six nonduplicate S. epidermidis isolates | From definite PICC-CRBSI episodes | Study-hospital microbiology laboratory | SE-01 to SE-06 | Strain heterogeneity |
| Susceptibility-control strain | Staphylococcus aureus | CLSI quality-control strain | ATCC | 29213 | MIC control |
| Cell line | THP-1 human monocytes | Human monocytic cell line | ATCC | TIB-202 | Macrophage model |
| Berberine | Berberine chloride | Purity ≥98% | Sigma-Aldrich | B3251 | Biofilm and cell exposure |
| Solvent | Dimethyl sulfoxide | Cell-culture grade | Sigma-Aldrich | D2650 | Vehicle |
| Bacterial media | Tryptic soy broth; tryptic soy agar; cation-adjusted Mueller–Hinton broth | Microbiology grade | BD Difco | 211825; 236950; 212322 | Growth, enumeration, MIC |
| Biofilm reagents | D-(+)-glucose; crystal violet; phosphate-buffered saline | Standard microbiology reagents | Sigma-Aldrich/Gibco | G8270; C0775; 10010023 | Biofilm formation, biomass, washing |
| Cell-culture reagents | RPMI-1640, fetal bovine serum, penicillin–streptomycin, 2-mercaptoethanol, PMA | Cell-culture grade | Gibco/Sigma-Aldrich | 11875093; 10082147; 15140122; 21985023; P8139 | THP-1 culture and differentiation |
| ELISA and viability assays | Human IL-6 ELISA; Human TNF-α ELISA; Cell Counting Kit-8 | Commercial kits | R&D Systems/Dojindo | D6050; DTA00D; CK04 | Cytokine and viability assays |
| Western blot reagents | RIPA buffer, inhibitor cocktail, BCA kit, phospho-p65, total p65, β-actin, HRP secondary antibody, PVDF membrane, ECL substrate | Commercial reagents | CST/Thermo Fisher/Merck/Bio-Rad | 9806; 5872; 23225; 3033; 8242; 4970; 7074; IPVH00010; 1705061 | NF-κB pathway analysis |
| Instruments/software | Multimode plate reader, sonication bath, chemiluminescence imager, Image Lab, R | Standard laboratory instruments/software | Molecular Devices/Branson/Bio-Rad/R Foundation | SpectraMax iD5; Bransonic M1800H; ChemiDoc MP; Image Lab 6.1; R 4.4.2 | Measurement and analysis |
| Microscopy platform | SEM or confocal microscope | Used only for representative biofilm images when available | Institutional imaging core | SEM or confocal model | Descriptive visualization |

B. PICC-coupon biofilm experiment

| **Component** | **Specification** |
| --- | --- |
| Bacterial strains | ATCC 35984 and six nonduplicate clinical S. epidermidis isolates |
| Species rationale | S. epidermidis was selected because coagulase-negative staphylococci were common in the clinical cohort and because S. epidermidis is a canonical catheter-biofilm species. The experiment did not model Gram-negative or fungal PICC-CRBSI. |
| Initial inoculum | Approximately 1 × 10⁶ CFU/mL |
| Biofilm medium | Tryptic soy broth with 1% glucose |
| Coupon material | Unused sterile 10-mm polyurethane PICC segment |
| Exposure conditions | Vehicle, 1/8 MIC, 1/4 MIC, and 1/2 MIC |
| Incubation | Static incubation at 37°C for 24 h |
| Primary endpoint | log₁₀ CFU per coupon |
| Secondary endpoints | Crystal violet OD590, planktonic OD600, and biomass-to-growth ratio |
| Descriptive imaging | Representative SEM or confocal images when available; not a quantitative endpoint |
| Technical replication | Three coupon wells per strain–dose–endpoint condition |
| Independent replication | Three independent experimental runs |
| Analysis unit | Mean of three technical wells for each strain–dose–run condition |
| Analytical observations | 21 strain–run means per exposure level |
| Controls | Vehicle growth control, sterile coupon background control, and concentration-matched berberine optical blank |

C. Strain-specific berberine concentrations and antimicrobial susceptibility profiles

| **Strain** | **Berberine MIC, µg/mL** | **1/8 MIC** | **1/4 MIC** | **1/2 MIC** | **Oxacillin** | **Vancomycin** | **Linezolid** | **Daptomycin** | **Rifampicin** | **Clindamycin** |
| --- | --- | --- | --- | --- | --- | --- | --- | --- | --- | --- |
| ATCC 35984 | 128 | 16 | 32 | 64 | Susceptible | Susceptible | Susceptible | Susceptible | Susceptible | Susceptible |
| SE-01 | 64 | 8 | 16 | 32 | Resistant | Susceptible | Susceptible | Susceptible | Susceptible | Resistant |
| SE-02 | 128 | 16 | 32 | 64 | Resistant | Susceptible | Susceptible | Susceptible | Resistant | Resistant |
| SE-03 | 128 | 16 | 32 | 64 | Susceptible | Susceptible | Susceptible | Susceptible | Susceptible | Susceptible |
| SE-04 | 256 | 32 | 64 | 128 | Resistant | Susceptible | Susceptible | Susceptible | Resistant | Resistant |
| SE-05 | 64 | 8 | 16 | 32 | Resistant | Susceptible | Susceptible | Susceptible | Susceptible | Resistant |
| SE-06 | 256 | 32 | 64 | 128 | Resistant | Susceptible | Susceptible | Susceptible | Resistant | Resistant |

D. Biofilm-conditioned medium

| **Item** | **Specification** |
| --- | --- |
| Source biofilm | Untreated 24-h ATCC 35984 biofilm on polyurethane PICC coupons |
| Conditioning medium | Serum-free RPMI-1640 |
| Conditioning duration | 4 h at 37°C |
| Clarification | 3,000 × g for 10 min |
| Sterilization | 0.22-µm membrane filtration |
| Sterility control | No colony growth after 48-h culture on tryptic soy agar |
| Storage | Single-use aliquots at −80°C |
| Independent preparations | Three |

E. Plasma-conditioned macrophage experiment

| **Component** | **Specification** |
| --- | --- |
| Plasma source | Individual lithium-heparin plasma collected before PICC insertion |
| Donor groups | 12 low-inflammatory-risk and 12 high-inflammatory-risk donors |
| Low-risk definition | Combined-model risk <5% and biomarker-model risk ≤4.1% |
| High-risk definition | Combined-model risk ≥15% and biomarker-model risk ≥13.8% |
| Matching | Frequency matching by sex and 5-year age category |
| Plasma and conditioned medium | 10% plasma + 10% sterile biofilm-conditioned medium |
| Berberine concentrations | 0, 5, and 10 μM |
| Final dimethyl sulfoxide | 0.1% in all conditions |
| Clinical-achievability interpretation | Exploratory in vitro concentrations; not assumed to be clinically achievable systemic concentrations. |
| Cell density | 2 × 10⁵ THP-1 cells per well |
| Differentiation | PMA 100 ng/mL for 48 h followed by 24-h rest |
| Berberine pretreatment | 1 h |
| Collection times | NF-κB at 6 h; cytokines and viability at 24 h |
| Endpoints | IL-6, TNF-α, phospho-p65/total-p65 ratio, and cell viability |
| Technical replication | Duplicate wells per donor–dose condition |
| Biological replication | Individual donor plasma samples were biological replicates; plasma was not pooled. |
| Analysis unit | Mean of duplicate wells for each donor–dose condition |
| Plate design | Donor groups and berberine concentrations balanced across plates |
| Quality criteria | ELISA duplicate CV ≤15%; spike recovery 80%–120%; acceptable dilution parallelism; viability ≥85% of donor-matched vehicle |
| Controls | Cell-free optical controls and common reference lysate for western blot normalization |

Notes: MIC, minimum inhibitory concentration; PICC, peripherally inserted central catheter; CRBSI, catheter-related bloodstream infection; CFU, colony-forming units; ELISA, enzyme-linked immunosorbent assay; NF-κB, nuclear factor kappa B; PMA, phorbol 12-myristate 13-acetate. Lot numbers, cell passage numbers, isolate identifiers, susceptibility reports, plate identifiers, gel identifiers, preparation dates, microscopy image identifiers when available, and instrument quality-control records were retained.
